# *BpForms* and *BcForms*: a toolkit for concretely describing non-canonical polymers and complexes to facilitate global biochemical networks

**DOI:** 10.1186/s13059-020-02025-z

**Published:** 2020-05-18

**Authors:** Paul F. Lang, Yassmine Chebaro, Xiaoyue Zheng, John A. P. Sekar, Bilal Shaikh, Darren A. Natale, Jonathan R. Karr

**Affiliations:** 1 Icahn Institute for Data Science and Genomic Technology, Icahn School of Medicine at Mount Sinai, New York, 10029 NY USA; 2 Department of Genetics and Genomic Sciences, Icahn School of Medicine at Mount Sinai, New York, 10029 NY USA; 3 Department of Biochemistry, University of Oxford, South Parks Road, Oxford, OX1 3QU UK; 4 Institut de Génétique et de Biologie Moléculaire et Cellulaire, Institut National de la Santé et de la Recherche Médicale, Centre National de la Recherche Scientifique, Université de Strasbourg, Illkirch, 67404 France; 5 Protein Information Resource, Georgetown University Medical Center, Washington, DC, 20007 USA

**Keywords:** Format, Software, Macromolecule, Proteoform, Residue, Modification, Crosslink, Multiscale, Genome-scale, Network

## Abstract

Non-canonical residues, caps, crosslinks, and nicks are important to many functions of DNAs, RNAs, proteins, and complexes. However, we do not fully understand how networks of such non-canonical macromolecules generate behavior. One barrier is our limited formats for describing macromolecules. To overcome this barrier, we develop *BpForms* and *BcForms*, a toolkit for representing the primary structure of macromolecules as combinations of residues, caps, crosslinks, and nicks. The toolkit can help omics researchers perform quality control and exchange information about macromolecules, help systems biologists assemble global models of cells that encompass processes such as post-translational modification, and help bioengineers design cells.

## Background

A central goal in biology is to understand how all of the molecules and processes in cells generate behavior. The Central Dogma generates sequences of the standard four DNA nucleotides, four RNA nucleotides, and twenty amino acids linked by standard sugar-phosphate and peptide bonds. Beyond the Central Dogma, processes such as epigenetic, post-transcriptional, and post-translational modification; DNA damage and repair; and signal transduction involve additional chemistry including (a) thousands of non-canonical, or modified, DNA nucleotides, RNA nucleotides, and amino acids; (b) caps, or residues which can only be located at the end of a polymer because they can only bond with preceding or following residues; (c) crosslinks, or covalent bonds between non-adjacent residues such as disulfide bonds; and (d) nicks, or the absence of a covalent bond between adjacent residues, such as during the discontinuous replication of the lagging DNA strand. For example, prokaryotic restriction/modification systems use the non-canonical DNA residue N^6^-methyl-adenosine monophosphate to selectively degrade foreign DNA [1]; tRNAs use pseudouridine monophosphate to translate multiple codons [2]; and signaling cascades use phosphoserine, phosphothreonine, and phosphotyrosine to encode information into proteins [3]. Eukaryotic cells post-transcriptionally add 7-methylguanylate 5 ^′^ caps to stabilize their mRNA [4]. Disulfide bonds between distant cysteines help proteins fold [5], and DNA crosslinks are vital to the function of many anti-cancer drugs [6]. DNA nicks are a key feature of the discontinuous replication of the lagging DNA strand [7], vital to the control of the superhelicity of DNA by topoisomerases [8], and essential to DNA mismatch repair [9].

Recent technical advances have enabled detailed information about individual non-canonical DNA, RNA, and protein residues. For example, SMRT-seq can identify the locations of DNA methylations with single-nucleotide resolution [10] and mass spectrometry can identify hundreds of protein modifications [11]. Furthermore, several repositories have compiled extensive data about non-canonical residues and crosslinks in DNA [12–15], RNA [16, 17], and proteins [18–23], as well data about the subunit composition and crosslinks of complexes [21, 23–26]. Despite this progress, we still do not have an integrated understanding of epigenetic modification, post-transcriptional modification, or post-translational modification, much less a global understanding of entire cells.

Whole-cell (WC) models [27, 28], which aim to predict phenotype from genotype by representing all of the biochemical activity in cells, are a promising tool for integrating diverse information about macromolecules into a holistic understanding of cells. However, it remains challenging to build global biochemical networks, such as whole-cell models, because we have few tools for capturing the structures of non-canonical macromolecules and linking them together into networks. For example, formats such as BioNetGen [29] and the Systems Biology Markup Language (SBML) [30] are cumbersome for modeling post-transcriptional modification because they have limited capabilities to represent the structure of RNA [31, 32].

Representations of the primary structures of macromolecules that can be combined with modeling frameworks such as SBML would provide a significant step toward global biochemical networks. Combined with software tools for interpreting their semantic meaning, such representations could also facilitate the curation, exchange, and quality control of structural information about macromolecules for a wide range of omics and systems and synthetic biology research.

Several formats have limited abilities to represent the primary structures of non-canonical macromolecules. Molecular formats which represent atoms and bonds, such as the International Chemical Identifier (InChI) [33], the PDB format [34], the Simplified Molecular-Input Line-Entry System (SMILES) [35], and the BigSMILES [36], can represent non-canonical residues, 5 ^′^ caps, crosslinks, and nicks. However, their verbosity makes them cumbersome for network research. Omics and systems biology formats, such as BioPAX [37], the Biological Expression Language (BEL) [38], the IUPAC/IUBMB notation [39] (often associated with the FASTA format), the MODOMICS nomenclature [16], Proteomics Standards Initiative Extended FASTA Format (PEFF) [40], the PRO notation [22], ProForma [41], and the Synthetic Biology Open Language (SBOL) [42], use coarser representations that are conducive to network research. However, these formats have limited abilities to represent non-canonical residues, 5 ^′^ caps, crosslinks, and nicks, and they do not concretely represent the structures of molecules. Although HELM [43] supports concrete, high-level descriptions of polymers as sequences of named residues, HELM does not support high-level, named descriptions of crosslinks or nicks; HELM does not support high-level descriptions of complexes as bags of subunits; and HELM cannot identify missing or uncertain knowledge, which is essential for biological research.

**Fig. 1:**
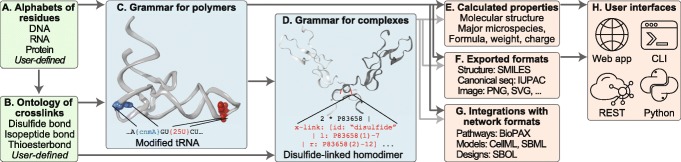
*BpForms*’ and *BcForms*’ representation of the primary structures of polymers and complexes. For example, *BcForms* represents a disulfide-linked dimer of the selenocysteine-modified tripeptide ACU (**a**, green box) as a set of polymeric subunits and crosslinks (**c**, green text), *BpForms* represents each tripeptide (**b**, blue boxes) as a sequence of residues (**d**, blue text), the protein alphabet represents the molecular structure of each residue and the atoms in each residue which are involved in bonds with adjacent residues (**e**, white boxes, green lines, and orange and blue letters), and the ontology of crosslinks represents the atoms involved in each crosslink (**f**, red line and red letters). *BpForms* uses SMILES to represent the molecular structure of each residue. The blue, black, and gray numbers illustrate the coordinate of each subunit, residue, and atom, respectively

Toward biochemical networks of entire cells, we developed *BpForms*-*BcForms*, an open-source toolkit for representing the primary structure of polymers and complexes with the precision of fine-grained formats such as SMILES and the brevity of coarse-grained formats such as the IUPAC/IUBMB format. *BpForms* includes extensible alphabets of hundreds of DNA, RNA, and protein residues (lists of the codes and structures of residues); an ontology of common crosslinks (list of the codes and structures of crosslinks); and a human- and machine-readable grammar (text format) for describing polymers as combinations of residues (including caps), backbone bonds between adjacent residues, intra-chain crosslinks, and nicks. *BpForms* describes polymers as combinations of residues, backbone bonds, crosslinks, and nicks (Fig. 1b, d) because this representation can capture any DNA, RNA, or protein and it is intuitive to many biologists. *BcForms* includes a human- and machine-readable grammar for combining polymers, small molecules, and inter-chain crosslinks into complexes (Fig. 1a, c).

Both tools include software for validating descriptions of macromolecules, calculating properties of macromolecules such as their formula, visualizing macromolecules, and exporting macromolecules to molecular formats such as SMILES. Both tools are available as a web application, REST API, command-line program, and Python library.

While the toolkit was motivated by whole-cell modeling, the toolkit also addresses several problems in transcriptomics, proteomics, systems biology, and synthetic biology. First, the toolkit can help omics researchers describe, exchange, quality control, and integrate information about post-transcriptional and post-translational modification. For example, as described below, we have used the toolkit to systemize the representation of the proteoforms in the Protein Ontology (PRO), a database of proteoforms, [22] and detect and correct errors in the database. The toolkit also makes it easier for the PRO Consortium to merge information from other resources to expand the database. Similarly, the toolkit can help modelers and bioengineers communicate the semantic meaning of models and genetic designs, making them easier to understand, reuse, and combine. In particular, the toolkit can help bioengineers communicate designs that involve modified genetic codes [44]. Second, the toolkit can help modelers construct multiscale models of cellular biochemistry. This includes systematically identifying gaps in models, correcting element imbalances, and merging models of multiple pathways. Toward whole-cell models, below, we illustrate how the toolkit can help modelers merge models of multiple signaling cascades and metabolism. Third, the toolkit makes it easier for bioengineers to learn and communicate design constraints on transforming parts into alternative hosts. Together with a parts repository such as SynBioHub [45], the toolkit could be used to develop a dependency management system for biological parts analogous to the Advanced Package Tool (APT) [46] for Ubuntu, which could help bioengineers design genetic circuits.

Here, we describe the toolkit and demonstrate how it can facilitate omics, modeling, and synthetic biology. First, we describe the toolkit, including the alphabets of residues, the ontology of crosslinks, the grammars, and the software tools. Second, we describe how the toolkit can be integrated into formats for networks such as BioPAX, CellML [47], SBML, and SBOL to describe the macromolecules involved in pathways, models, and genetic designs. Next, we describe the advantages of the toolkit over existing formats and other resources for omics, systems biology, and bioengineering. Lastly, we present multiple case studies that illustrate how the toolkit can help researchers describe, quality control, exchange, and integrate diverse information about macromolecules into networks such as protein-protein networks. Ultimately, we anticipate that the toolkit will facilitate whole-cell models.

## Results

### Toolkit for concisely representing non-canonical polymers and complexes

**Fig. 2:**
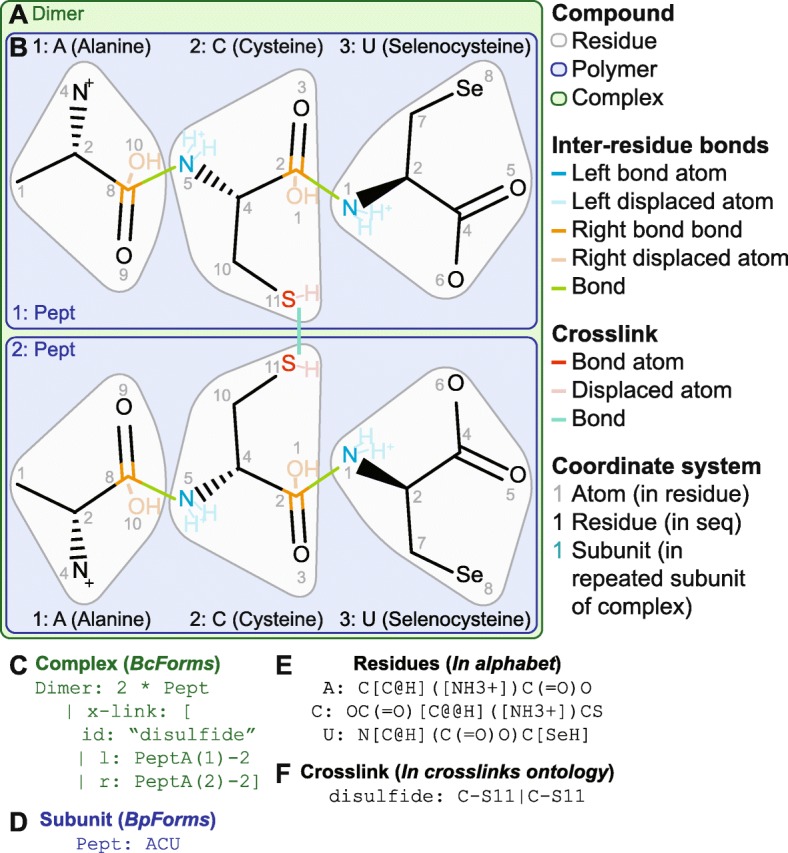
Overview of the *BpForms*-*BcForms* toolkit. The toolkit includes (**a**) extensible alphabets of DNA, RNA, and protein residues and 5 ^′^ caps; (**b**) an ontology of crosslinks; (**c**) a grammar for composing polymers from residues, 5 ^′^ caps, crosslinks, and nicks; (**d**) a grammar for composing complexes from polymers and crosslinks; software tools for validating descriptions of macromolecules, (**e**) calculating molecular properties of macromolecules, (**f**) exporting macromolecules to other formats and visualizing macromolecules; (**g**) protocols for integrating descriptions of macromolecules into omics, systems biology, and synthetic biology formats for networks, models, and genetic designs; and (**h**) multiple user interfaces. (**c**) The grammar (text) for describing polymers (image); the colored text illustrates how the grammar captures non-canonical residues (colored portions of the image). (**d**) The grammar (colored text) for describing complexes (image); the colored text illustrates how the grammar captures inter-subunit crosslinks (colored portion of image)

The *BpForms*-*BcForms* toolkit includes several interrelated tools for describing, validating, visualizing, and calculating properties of the primary structure of DNA, RNA, proteins, and complexes (Fig. 2). Here, we describe the components of the toolkit including the representations and grammars for polymers and complexes; the alphabets of residues; the ontology of crosslinks; the software tools for quality controlling, analyzing, and visualizing macromolecules; the protocols for integrating the toolkit with formats for network research; and the user interfaces.

#### Representation of the primary structure of polymers and complexes

*BpForms* and *BcForms* use three layers to describe the primary structures of polymers and complexes. (1) *BcForms* represents complexes (Fig. 1a, c) as a set of subunits, including their stoichiometries, and a set of crosslinks between the subunits. Subunits which are DNA, RNA, or protein polymers can be represented using *BpForms*; subunits which are small molecules, such as vitamins, can be represented using molecular formats such as SMILES. (2) *BpForms* represents polymers (Fig. 1b, d) as a sequence of residues and nicks, a set of crosslinks, and a Boolean indicator of circularity. (3) Residues are represented by their molecular structures and the atoms which are involved in bonds with adjacent residues. Crosslinks are also represented by the atoms involved in each crosslink.

##### Residues and caps

Each residue is represented by its molecular structure, a list of the atoms which can form bonds with adjacent residues, and a list of the atoms which are displaced by the formation of these bonds (Fig. 1e). These lists of atoms are optional to enable *BpForms* to represent internal and terminal nucleic and amino acids, including 5 ^′^ caps such as 7-methylguanylate, which eukaryotic cells add post-transcriptionally to stabilize mRNA. As described below, *BpForms* can also capture missing or uncertain information about residues, such as uncertainty about the precise location of a non-canonical residue. As described in Section 3.1 of Additional file 1, *BpForms* can also represent metadata about residues such as their names and references to related entries in RESID [19].

The toolkit uses a hybrid approach to separate the molecular details of each residue from the description of each macromolecule. The chemical details of common residues are encapsulated into three alphabets of DNA, RNA, and protein residues. Each alphabet is a collection that maps the code of each residue to its molecular details. In addition, users can create custom alphabets or define additional residues within descriptions of macromolecules. This hybrid approach standardizes the representation of common residues while enabling the toolkit to represent any residue.

##### Nicks

A nick is the absence of an inter-residue bond between successive residues such as a strand break in double-stranded DNA. While such macromolecules can also be described as complexes, nicks are a more natural representation in many cases. *BpForms* represents each nick by its position within the residue sequence of its parent polymer.

##### Crosslinks

Each crosslink is represented as lists of the atoms which form a bond between residues and the atoms which are displaced by the formation of these bonds (Fig. 1f). The toolkit uses a similar hybrid approach to separate the molecular details of crosslinks from the descriptions of macromolecules. Common crosslinks are encapsulated into an ontology, and users can create a custom ontology or define additional crosslinks within descriptions of macromolecules.

##### Coordinate system

The toolkit uses a hierarchical coordinate system to describe the atoms involved in each inter-residue bond and crosslink. The coordinate of each subunit consists of its id and an integer which ranges from one to the stoichiometry of the subunit in its parent complex. The coordinate of each residue is its position within the residue sequence of its parent polymer. The coordinate of each atom is its position within the canonical SMILES ordering of the atoms in its parent residue. Section 5 of Additional file 1 contains more information about these coordinates.

##### Uncertainty

*BpForms* and *BcForms* can represent several types of uncertainty about molecules. (a) To support mass spectrometry, *BpForms* can capture additional mass and charge which have been observed, but which cannot be interpreted as a specific residue. (b) *BpForms* can capture uncertainty about the location and chemical origin of residues. For example, *BpForms* can capture knowledge that a protein contains a phosphorylated tyrosine or threonine within a range of positions. (c) *BpForms* and *BcForms* can capture unstructured comments about each residue and crosslink.

#### Alphabets of DNA, RNA, and protein residues

To support a broad range of research, *BpForms* includes the most extensive alphabets of DNA, RNA, and protein residues to date. The DNA alphabet includes 422 deoxyribose nucleotide monophosphates and terminal ends derived from data about DNA damage and repair from REPAIREtoire [13], structural data from the Protein Data Bank Chemical Component Dictionary (PDB CCD) [48], and chemoinformatics data from DNAmod [12]. The RNA alphabet includes 378 ribose nucleotide monophosphates and 5 ^′^ caps derived from biochemical data from MODOMICS [49] and the RNA Modification Database [17] and structural data from the PDB CCD. The protein alphabet has 1435 amino acids and carboxy and amino termini derived from biochemical data from RESID [19] and structural data from the PDB CCD. The *BpForms* website contains pages which display the residues in each alphabet. Section 6 of Additional file 1 describes how we constructed the alphabets.

#### Ontology of crosslinks

To concisely describe the molecular structures of polymers and complexes, the toolkit includes an ontology of crosslinks. Currently, the ontology contains 36 common protein crosslinks, such as disulfide bonds, isopeptide bonds, and thioesters. Going forward, we encourage the community to contribute additional crosslinks, including DNA-DNA, RNA-RNA, DNA-protein, and RNA-protein crosslinks, by submitting GitHub pull requests or by contacting the authors. Section 8 of Additional file 1 describes how we plan to manage community contributions to *BpForms* and *BcForms*. The *BpForms* website contains a page that displays the crosslinks in the ontology. Section 7 of Additional file 1 describes how we constructed the ontology.

#### Textual grammars for describing polymers and complexes

Tables 1 and 2 illustrate the toolkit’s grammars for describing polymers and complexes, and Fig. 1 illustrates the chemical semantics of a homodimer encoded in the grammars. Section 3 of Additional file 1 and the *BpForms* and *BcForms* websites provide detailed descriptions of the grammars and additional examples. Section 4 of Additional file 1 contains formal descriptions of the grammars.

**Table 1:** Examples of the *BpForms* grammar for describing polymers

**Table 2:** Examples of the *BcForms* grammar for describing complexes

	

#### Syntactic and semantic validation of descriptions of macromolecules

To help quality control information about macromolecules, the toolkit can verify the syntactic and semantic correctness of macromolecules encoded in the grammars. First, the toolkit can verify that textual descriptions of macromolecules are syntactically consistent with the grammars and identify any errors. Second, the toolkit can verify that macromolecules described with the grammars are semantically consistent and identify any errors. For example, the toolkit can identify pairs of adjacent amino acids that cannot form peptide bonds because the first amino acid does not have a carboxy-terminus, or because the second amino acid does not have an amino terminus. Section 9 of Additional file 1 details the semantic validations implemented by the toolkit. We anticipate that these quality controls will help researchers exchange reliable information and assemble this information into high-quality networks.

#### Analyses of polymers and complexes

The toolkit can calculate properties of molecules such as their major protonation and tautomerization states, chemical formula, molecular weight, and charge. We have begun to use these properties to quality control models. For example, we are using the chemical formulae to verify that each reaction is element-balanced, including reactions that represent transformations of macromolecules such as post-transcriptional modifications.

The toolkit can also compare macromolecules to determine their equality or differences. We plan to use this feature to implement automated procedures for merging models with shared species.

#### Molecular and sequence visualizations

**Fig. 3:**
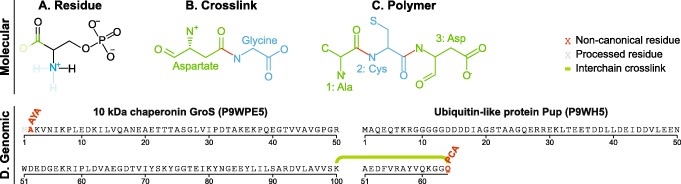
*BpForms* and *BcForms* can generate molecular and genomic visualizations of macromolecules. *BpForms* can generate molecular visualizations of residues such as phosphoserine (**a**), crosslinks such as an isoaspartyl glycine isopeptide bond (**b**), and polymers such as the tripeptide ACD (**c**). The blue and green letters in **a** indicate the atoms which can bond with preceding and following residues; the light blue and green letters indicate the atoms which are displaced by the formation of these bonds. The blue and green elements in **b** indicate the individual residues involved in the crosslink; the red line indicates the covalent bond that crosslinks the residues. The green elements in **c** indicate the first and third residues in the peptide, the blue elements indicate the second residue, and the red lines indicate the covalent bonds between the residues. *BpForms* and *BcForms* can also generate sequence-based visualizations of polymers and complexes such as the pupylation of chaperonin GroS (**d**). The left and right tracks indicate the canonical residues of GroS and Pup, respectively. The gray letters indicate the residues which are removed post-translationally. The horizontal red letters indicate the residues which are post-translationally modified. The rotated red letters indicate the type of each post-translational modification. The green line indicates the residues which are post-translationally crosslinked

To help analyze macromolecules, the toolkit can generate molecular and sequence visualizations of residues, 5 ^′^ caps, crosslinks, polymers, and complexes. The molecular visualizations display each atom and bond and use colors to highlight features such as individual residues, inter-residue and crosslink bonds, and the atoms that are displaced by the formation of the inter-residue bonds (Fig. 3a–c). The molecular visualizations can also display the coordinate of each residue and atom. The sequence visualizations include interactive tooltips that describe each non-canonical residue, crosslink, and nick (Fig. 3d).

#### Export to other molecular and sequence formats

For compatibility with structural and biochemical research, the toolkit can export macromolecules to molecular formats such as the PDB format. For compatibility with genomics research, the toolkit can also generate canonical IUPAC/IUBMB sequences for polymers and export multiple polymers to FASTA documents [50].

#### Integration with frameworks for network-scale research

The toolkit can facilitate network-scale research through integration with omics and systems and synthetic biology frameworks such as BioPAX, CellML, SBML, and SBOL. Section 10 of Additional file 1 illustrates how the toolkit can be incorporated into these frameworks.

#### User interfaces

The toolkit includes four interfaces: a web application, a REST API, a command-line program, and a Python library. Section 11 of Additional file 1 contains examples of the interfaces.

### Comparison with existing formats and alphabet-like resources

We believe that the toolkit is well-suited for network research because it improves upon several existing resources for representing polymers and complexes. The toolkit (a) introduces representations for crosslinks and nicks; (b) contains the most extensive alphabets of DNA, RNA, and protein residues to date; (c) introduces an ontology of concrete crosslinks; (d) uses a novel combination of ontologies and inline definitions of residues and crosslinks to standardize the representation of common residues and crosslinks while being capable of representing any residue or crosslink; (e) includes a novel coordinate system that makes it easy to address specific atoms in macromolecules; and (f) includes more extensive quality controls for descriptions of macromolecules. Here, we outline how the toolkit improves upon several existing resources for representing polymers and complexes.

#### Comparison of *BpForms* with existing formats for polymers

*BpForms* represents the primary structures of DNAs, RNAs, and proteins as combinations of residues, caps, crosslinks, nicks, and circularity. In contrast, molecular formats such as SMILES are cumbersome for polymers and coarse-grain formats such as ProForma and network formats such as BioPAX do not concretely represent molecules. *BpForms* also combines the features of previous fine- and coarse-grain formats: it can capture missing information similar to ProForma, it is human-readable like other coarse-grain formats, it is machine-readable like molecular formats, it is composable with network formats such as SBML like molecular formats, and it is backward compatible with the IUPAC/IUBMB format like other coarse-grain formats. Section 13.1 of Additional file 1 and Table S1 provide a detailed comparison of *BpForms* with several other formats.

#### Comparison of *BpForms* alphabets with existing databases

The *BpForms* alphabets are the most extensive alphabets of DNA, RNA, and protein residues because they incorporate structural, biochemical, and physiological data from several sources. Along with the PDB CCD, the *BpForms* alphabets are also the only alphabets that consistently represent DNA, RNA, and protein residues and which represent the inter-residue bonding sites of each residue, enabling residues to be combined into concrete molecular structures. In contrast, DNAmod, REPAIRtoire, MODOMICS, RESID, and the RNA Modification Database each only represent either DNA, RNA, or protein residues; the residues in DNAmod, REPAIRtoire, MODOMICS, and the RNA Modification Database are hard to compose into polymers because they represent nucleobases and nucleosides rather than nucleotides; and DNAmod, REPAIRtoire, MODOMICS, RESID, and the RNA Modification Database do not capture sites. Section 13.2 of Additional file 1 and Table S2 provide a detailed comparison of the *BpForms* alphabets with these resources.

#### Comparison of the *BpForms* crosslinks ontology with existing resources

Several resources contain information about crosslinks. In particular, the UniProt-controlled vocabulary of post-translational modifications contains textual descriptions of over 100 types of crosslinks. However, UniProt does not describe the molecular structures of the crosslinks. MOD, REPAIRtoire, and RESID also indirectly represent crosslinks by representing dimers and trimers that contain crosslinks.

In contrast, the *BpForms* ontology directly represents the chemical structures of crosslinks. This enables crosslinks to be composed into concrete structures. Section 13.3 of Additional file 1 and Table S3 provide a detailed comparison of the *BpForms* crosslinks ontology with these resources.

#### Comparison of *BcForms* with existing formats for complexes

Despite their importance, only a few formats can represent complexes. The PDB format can capture the 3-dimensional structures of complexes. BioPAX and SBOL can capture the subunit composition of complexes. *BcForms* improves upon BioPAX and SBOL by capturing the primary structures of complexes, including the stoichiometry of each subunit and crosslinks. *BcForms* improves upon the PDB format by providing a more concise, human-readable format that can be composed with formats for networks such as SBML. Section 13.4 of Additional file 1 and Table S4 provide a detailed comparison of *BcForms* with several other formats.

### Case studies

We believe that the *BpForms*-*BcForms* toolkit can advance a wide range of omics and systems and synthetic biology research. Here, we illustrate how we have used the toolkit to improve the quality of the Protein Ontology (PRO) of modified forms of proteins; analyze the flux of tRNA modification in *Escherichia coli*; refine, expand, and compose a model of MAPK signaling with models of other pathways; and identify constraints on designing new strains of *E. coli*. Although some of the analyses could have been conducted without the toolkit, *BpForms* makes such analyses easier and more accessible to a wider range of investigators.

#### Proteomics: quality control of the protein ontology

One of the goals of proteomics is to characterize the proteoforms, or modified forms of proteins such as multi-phosphorylated states [51], in cells. Toward this goal, the PRO Consortium has integrated several different types of data into PRO, a database of over 7000 proteoforms. Because the consortium constructs PRO, in part, by hand, automated quality controls could help the consortium identify and correct errors.

To quality control PRO, first we encoded each applicable entry in PRO into the *BpForms* grammar and used the *BpForms* software to validate them. This identified several types of syntactical and semantic errors. For example, we identified proteoforms that refer to sites with coordinates greater than the length of the parent sequence. We also identified modified residues whose structures are inconsistent with the translated sequences of their parent proteins, such as a phosphorylated serine annotated at the position of a tyrosine. Investigation of these errors revealed that some were imported into PRO when other resources were merged into PRO, some were due to different coordinate systems used to describe modifications to processed proteins than that of the reference sequence (for example, papers that report positions relative to the translated sequence rather than to the sequence after the removal of the initiator methionine), and some were due to not updating the positions of modifications when the reference sequences were corrected. We have corrected all of these errors in PRO. Our corrections will be released with the next version of PRO, 60.0.

To help the consortium continue to use the toolkit to quality control PRO, we developed a script that automates this analysis. Going forward, the consortium also plans to use *BpForms* to visualize proteoforms, automatically import proteoforms from external resources, and export proteoforms in *BpForms* format to help users use the PRO data in their research.

#### Systems biology: analysis of the flux of prokaryotic tRNA modification

To achieve whole-cell models, we must integrate information about all of the processes in cells and their interactions. Here, we illustrate how *BpForms* can help integrate information about the interaction between the RNA modification and metabolism of *E. coli* and identify gaps in models.

**Fig. 4:**
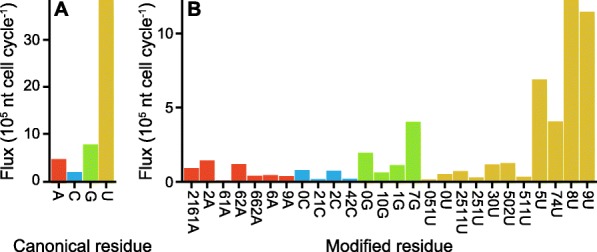
The *BpForms*-*BcForms* toolkit can facilitate integrative analyses of global intracellular networks. For example, we used *BpForms* to estimate the flux of tRNA modification in *E. coli* by canonical residue (**a**) and modified residue (**b**) from information about the modification, abundance, and turnover of each tRNA

First, we estimated the abundance of each tRNA from their total observed abundance [52, 53] and the observed relative abundance of each tRNA [54]. Second, we estimated the synthesis rate of each tRNA from its estimated abundance, the observed half-life of tRNA^Asn^ [55], and the observed doubling time of *E. coli* [56]. Third, we used *BpForms* to interpret the post-transcriptionally modified sequence of each tRNA curated by MODOMICS [16]. Fourth, we estimated the total synthesis rate of each modification from the synthesis rate and modification of each tRNA (Fig. 4).

This analysis revealed that *E. coli* tRNAs contain 26 types of modified residues that are collectively synthesized approximately 5.2 million times per cell cycle, that the five most frequent modifications account for 73.8% of all modifications, and that uridine modifications account for 55.0% of all modifications. Next, we tried to use a metabolic model to analyze how *E. coli* recycles these modified nucleic acids following RNA degradation. However, we found that even the iML1515 model [57], one of the most comprehensive models of cellular metabolism, only represents the free form of one of the modified residues (9U, pseudouridine). Therefore, metabolic models must be expanded to capture the recycling of modified nucleic acids. The reaction networks of these models must also be expanded to encompass the production of the donors for the modifications, such as S-sulfanyl-L-cysteine, the sulfur donor for 4-thiouridine (74U). Taken together, *BpForms* helped us to evaluate the global flux of *E. coli* tRNA modification, as well as identify gaps in metabolic models to understanding the metabolic impact of RNA modification.

#### Systems biology: systematic identification of gaps in the Kholodenko model of MAPK signaling

The Kholodenko model of MAPK signaling [58] describes several aspects of how the pathway transduces extracellular signals for growth, differentiation, and survival into the phosphorylation of MAPK. However, the model does not account for several other factors which could impact how the pathway transduces signals in disease states, such as the expression of the enzymes involved in the pathway, the regulation of the activity of these enzymes by related signaling pathways, and the availability of GTP for phosphorylation which can be diminished in starvation conditions.

**Fig. 5:**
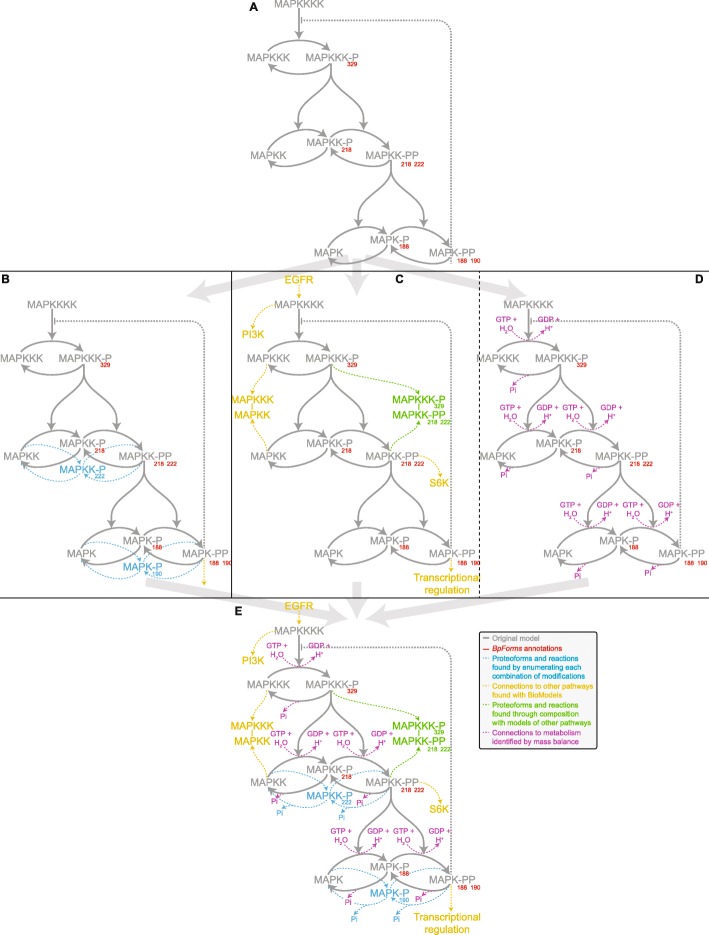
The *BpForms*-*BcForms* toolkit can help modelers refine, expand, and compose global intracellular networks. For example, we used *BpForms* to systematically identify ways to improve and expand the Kholodenko model of MAPK signaling to capture signaling under a broader range of conditions (**a**, gray) by using *BpForms* to capture the semantic meaning of each species (**a**, red), identify missing protein states (**b**, blue), identify other models that represent similar proteins which could be composed with the Kholodenko model (**c**, yellow) which could reveal additional missing combinations of species (**c**, green), and identify element imbalances which indicate missing metabolites which could facilitate composition with metabolic models (**d**). Together, this could enable a substantially more comprehensive and predictive model that can explain eukaryotic signaling under a broader range of disease conditions (**e**)

Toward a more comprehensive understanding of eukaryotic signaling across a broader range of conditions, we used *BpForms* to systematically identify gaps in the Kholodenko model and opportunities to merge the model with models of other pathways. To enable this analysis, we first obtained an SBML-encoded version of the model, manually curated the specific proteoforms indicated by the brief protein names provided with the published model, encoded these proteoforms into *BpForms* (Fig. 5a), and embedded these *BpForms* representations into the SBML representation of the model. We had to do this manually because Kholodenko did not report this information. We believe that the *BpForms* annotations make the model more semantically precise and understandable.

First, we used the *BpForms* annotations to systematically identify missing proteoforms that could help the model explain how the MAPK pathway transduces signals. Specifically, we used *BpForms* to identify two missing combinations of the individual protein modifications represented by the model and as many as four missing reactions that involve these species (Fig. 5b). These additional species and reactions could help the model better capture the kinetics of MAPKK and MAPKKK activation and deactivation and, in turn, better capture how the pathway transduces signals.

Next, we used the *BpForms* annotations to identify opportunities to merge the Kholodenko model with models of other signaling cascades. Specifically, we searched BioModels for other models that represent similar proteoforms. This analysis identified several models that represent EGFR, PI3K, S6K, and the transcriptional outputs of the MAPK pathway that could be composed with the Kholodenko model. Furthermore, this combination of models enabled us to identify emergent combinations of proteoforms that are missing from the individual models (Fig. 5c).

Lastly, to identify opportunities to merge the Kholodenko model with a model of metabolism, we used the *BpForms* annotations to systematically identify unbalanced reactions with missing metabolites. This analysis identified four missing species that, if added to the Kholodenko model, would make the model composable with models of metabolism (Fig. 5d).

Taken together, *BpForms* can be used to create more comprehensive, detailed, understandable, and composable models by helping researchers systematically describe the semantic meaning of models, fix imbalances in models, and identify gaps in models.

Going forward, we envision combining *BpForms* and *BcForms* with automated model merging tools such as semanticSBML [59] and SemGen [60] to provide researchers more powerful tools for merging models. Without formats for clearly describing the semantic meaning of each model component, these tools currently have to infer the meaning of each component from its network context, which is imperfect, limiting their capabilities to merge models. By enabling clear descriptions of the semantic meaning of each model component, *BpForms* and *BcForms* would make it easier for these tools to identify common components across models and correctly merge models.

#### Synthetic biology: systematic identification of design constraints

A promising way to engineer cells is to combine naturally occurring parts, such as genes that encode metabolic enzymes, in an accommodating host, such as *E. coli*. However, there are numerous potential barriers to transforming parts into other cells. For example, parts that contain post-translational modifications may not be functional in cells that cannot synthesize these modifications. While the literature contains substantial information about such dependencies, this information is not readily available to bioengineers for genetic design because we have limited tools for describing this information, and the information is scattered across the literature. Here, we illustrate how the synthetic biology community could use *BpForms* to describe and leverage these dependencies.

First, we developed custom codes to learn constraints on the transformation of parts into *E. coli* from the PDB. (1) We identified the modifications that have been observed in *E. coli*. (2) We identified modifications that have not been observed in *E. coli* and the proteins which contain them. For example, we found that proteins that contain 4-hydroxproline (PDB CCD: HYP), such as collagen (UniProt: P02452), potentially cannot be transformed into *E. coli*. (3) We used the literature to confirm that *E. coli* cannot synthesize these modifications [61–63]. Table S5 lists the most common modifications which could constrain the transformation of proteins into *E. coli*.

The synthetic biology community could use *BpForms* to systematically describe these dependencies and make this knowledge broadly available to bioengineers for genetic design. The community could (1) use *BpForms* to describe parts for synthetic organisms, which would provide information about the post-transcriptional and post-translational modifications required for each part; (2) incorporate this information into parts repositories such as SynBioHub [45]; and (3) develop software tools for using the *BpForms* annotations in these repositories to automatically predict the additional machinery that must be co-transformed for a part to be functional in a new host. This would enable these repositories to function as dependency management systems for synthetic organisms, analogous to the Advanced Package Tool (APT) for Ubuntu packages. In turn, such dependency management systems could help bioengineers develop genetic designs more reliably.

## Discussion

### Extending the toolkit to support additional use cases

We developed the toolkit to help researchers concretely represent DNAs, RNAs, proteins, and complexes. To support a broader range of uses, we hope to expand the toolkit to meet additional needs. (a) Based on rarefaction analysis of the PDB, biology likely employs many more residues than represented by our alphabets. To capture a broader range of macromolecules, we plan to periodically import additional residues from the PDB CCD and other sources, as well as solicit additional residues from the community. Section 8 of Additional file 1 describes how we plan to manage community contributions to *BpForms* and *BcForms*. (b) In collaboration with the proteomics community, we hope to expand the crosslinks’ ontology to capture a broader range of proteins. (c) To make it easier to use custom alphabets and ontologies to exchange information about polymers, it may be helpful to develop registries of alphabets and ontologies, tools for merging alphabets and ontologies, tools for comparing polymers described with different alphabets and ontologies, and tools for migrating descriptions of polymers between alphabets and ontologies. Because the goal of this would be to facilitate interoperation among the community, we hope to get input from the community about how to best address these issues. (d) We hope to collaborate with other researchers to extend the toolkit to capture additional types of uncertainty, such as uncertainty in the positions of crosslinks, as well as to formalize the chemical semantics of the uncertainty metadata. (e) As the grammar and ontologies evolve over time, to make it easy to determine the versions of the grammar and ontologies used to describe a molecule, the grammar should be extended to capture its own version number and the version numbers of the referenced ontologies. The software tools should also be expanded to include the capability to migrate descriptions of molecules between successive versions of the grammars and ontologies.

### Community adoption as a common toolkit

Realizing the full potential of *BpForms* and *BcForms* as formats for the structures of macromolecules will require acceptance by the omics, systems biology, and synthetic biology communities. We have begun to solicit users by submitting the grammars to the EMBRACE Data And Methods (EDAM) ontology of formats, contributing the alphabets of residues and the ontology of crosslinks to BioPortal, proposing a protocol for using *BpForms* with SBOL, helping the PRO Consortium use *BpForms*, and encouraging the developers of central repositories of DNA, RNA, and protein modifications to export their data in *BpForms* format. We also plan to stimulate discussion among the BioPAX, CellML, and SBML communities about formalizing our integrations of the toolkit with their formats. To help developers incorporate the toolkit into software tools, we also plan to help developers generate parsers for the grammars for other languages.

### Community adoption as standards

Because the toolkit aims to help researchers exchange information, we believe that the alphabets of residues, the ontology of crosslinks, and the grammars should ultimately become community standards. To start, we encourage the community to contribute to the toolkit via GitHub pull requests. Going forward, we would like these resources to be governed by the community through an organization such as the Computational Modeling in Biology Network (COMBINE) [64].

### Enabling whole-cell models by combining the concreteness of chemistry with the extensibility of informatics

The toolkit achieves concrete and concise descriptions of macromolecules by combining a precise grammar with ontologies of residues and crosslinks. This hybrid approach helps researchers create chemically concrete descriptions of macromolecules from a broad range of data. Similar hybrid physiochemical-informatics approaches are needed to help researchers build physically concrete whole-cell models from a broad range of data and concisely describe these models.

### Toward multiscale models that utilize structural information

We have begun to use the toolkit to describe the chemical semantics of the species represented by network models. Going forward, we also plan to use the toolkit to help network models capture finer-grained mechanisms that involve combinatorial interactions, such as how methylation impacts transcription factor-DNA binding. To do this, we are developing a generalized rule-based modeling framework that encapsulates properties such as primary structures into species and links these properties to reactions. We anticipate that this framework, together with the toolkit, will make it easier to build fine-grained kinetic models of complex processes such as transcriptional backtracking, ribosomal queuing, and tmRNA ribosomal rescuing and combine them into whole-cell models.

## Conclusions

The *BpForms*-*BcForms* toolkit concisely represents the primary structure of macromolecules, including non-canonical residues, 5 ^′^ caps, crosslinks, and nicks, as well as several types of missing information. Furthermore, the toolkit standardizes the representation of common residues and crosslinks while extensibly accommodating any residue and crosslink by supporting both centrally and user-defined residues and crosslinks. The toolkit includes the most extensive alphabets of DNA, RNA, and protein residues to date; a chemically concrete ontology of crosslinks; an intuitive coordinate system for macromolecules; human and machine-readable grammars for macromolecules; and user-friendly software interfaces. The toolkit is backward compatible with the IUPAC/IUBMB and SMILES formats to maximize compatibility with existing tools. The toolkit can also be integrated with frameworks for network research such as BioPAX, CellML, SBML, and SBOL.

We anticipate that the toolkit will be a valuable tool for omics, systems biology, and synthetic biology. First, the tools can help researchers precisely communicate information about forms of macromolecules. Similarly, the tools can make models and genetic designs more understandable by capturing the semantic meaning of the species represented by models and the parts of synthetic organisms. For example, *BpForms* could describe proteins produced by expanded genetic codes.

The tools can also help quality control information about macromolecules. For example, the tools could help researchers find errors in reconstructed proteoforms, such as inconsistencies between the modified and translated sequences; merge duplicate entries in databases of proteoforms; and identify gaps and element imbalances in models.

In addition, the toolkit can help researchers integrate structural, epigenomic, transcriptomic, and proteomic information about macromolecules. For example, the tools can help researchers integrate observations of individual protein modifications into descriptions of entire proteoforms. The tools can also help researchers create integrated, multiscale models of entire cells by helping researchers link network models to structural information about each species, combine models, and fill gaps in models. Similarly, the tools can help bioengineers design cells by identifying parts that must be co-transformed with post-transcriptional and post-translational modification machinery.

## Methods

We designed *BpForms* and *BcForms* as separate tools to provide users light-weight tools for describing polymers and complexes. We implemented the toolkit using Python, ChemAxon Marvin, Flask-RESTPlus, Lark, Open Babel [65], YAML Ain’t Markup Language, and Zurb Foundation. Section 12 of Additional file 1 provides more information about the implementation.

## Supplementary information

**Additional file 1** Additional text, boxes, and tables. **Text.** Descriptions of the grammars, coordinate system, construction of the alphabets of residues and the ontology of crosslinks, semantic verification, user interfaces, and implementation; comparisons with other resources; glossary; and acronyms. **Box S1.** Integration of *BpForms* with the FASTA format to describe multiple polymers within a single document. **Box S2.** Integration of *BpForms* with BioPAX documents to describe the polymers involved in pathways. **Box S3.** Integration of *BpForms* with SBML to describe the semantic meaning of the macromolecules represented by models. **Box S4.** Integration of *BpForms* with CellML to describe the semantic meaning of components which represent macromolecules. **Box S5.** Integration of *BpForms* with SBOL to describe DNA, RNA, and protein parts. **Box S6.** Tutorial for the *BpForms* command line program. **Box S7.** Tutorial for the *BcForms* command line program. **Box S8.** Tutorial for the *BpForms* Python library. **Box S9.** Tutorial for the *BcForms* Python library. **Table S1.** Comparison between *BpForms* and other formats for describing polymers. **Table S2.** Comparison between *BpForms* and other collections of DNA, RNA, and protein residues. **Table S3.** Comparison between the *BpForms* crosslinks ontology and other resources that describe crosslinks. **Table S4.** Comparison between *BcForms* and other formats for describing complexes. **Table S5.** The most common constraints on transforming proteins into *E. coli* due to missing post-translational machinery learned from the PDB with *BpForms*. Most of the information in Additional file 1 is also in the online user and developer tutorials and documentations at https://bpforms.org, https://bcforms.org, https://sandbox.karrlab.org,and\url{https://docs.karrlab.org}.

**Additional file 2** Review history.
