# Supplementary material for: BpForms and BcForms: a toolkit for concretely describing non-canonical polymers and complexes to facilitate global biochemical networks
Source: Genome Biol. 2020 May 18;21:117. doi: 10.1186/s13059-020-02025-z (PMC7236495; doi:10.1186/s13059-020-02025-z)
Supplement: Supplementary file 1 — Additional file 1 Additional text, boxes, and tables. Text. Descriptions of the grammars, coordinate system, construction of the alphabets of residues and the ontology of crosslinks, semantic verification, user interfaces, and implementation; comparisons with other resources; glossary; and acronyms. Box S1. Integration of BpForms with the FASTA format to describe multiple polymers within a single document. Box S2. Integration of BpForms with BioPAX documents to describe the polymers involved in pathways. Box S3. Integration of BpForms with SBML to describe the semantic meaning of the macromolecules represented by models. Box S4. Integration of BpForms with CellML to describe the semantic meaning of components which represent macromolecules. Box S5. Integration of BpForms with SBOL to describe DNA, RNA, and protein parts. Box S6. Tutorial for the BpForms command line program. Box S7. Tutorial for the BcForms command line program. Box S8. Tutorial for the BpForms Python library. Box S9. Tutorial for the BcForms Python library. Table S1. Comparison between BpForms and other formats for describing polymers. Table S2. Comparison between BpForms and other collections of DNA, RNA, and protein residues. Table S3. Comparison between the BpForms crosslinks ontology and other resources that describe crosslinks. Table S4. Comparison between BcForms and other formats for describing complexes. Table S5. The most common constraints on transforming proteins into E. coli due to missing post-translational machinery learned from the PDB with BpForms. Most of the information in Additional file 1 is also in the online user and developer tutorials and documentations at https://bpforms.org, https://bcforms.org, https://sandbox.karrlab.org,and\url{https://docs.karrlab.org}. [file 13059_2020_2025_MOESM1_ESM.pdf]

BpForms and *BcForms*: Additional file 1, boxes S1–S9, and tables S1–S5

Paul F. Lang[1,2,3,*], Yassmine Chebaro[1,2,4,*], Xiaoyue Zheng[1,2,*], John A. P. Sekar[1,2], Bilal Shaikh[1,2], Darren A. Natale[5], and Jonathan R. Karr[1,2,**]

[1]Icahn Institute, Icahn School of Medicine at Mount Sinai, US
[2]Department of Genetics & Genomic Sciences, Icahn School of Medicine at Mount Sinai, US
[3]Department of Biochemistry, Oxford University, UK
[4]Institut de Génétique et de Biologie Moléculaire et Cellulaire, FR
[5]Protein Information Resource, Georgetown University Medical Center, US

[*]These authors contributed equally to this work
[**]Correspondence: karr@mssm.edu

Contents

1 Introduction **3**

2 Features of the *BpForms-BcForms* toolkit **3**

3 Overview of the grammars for polymers and complexes **4**
- 3.1 *BpForms* grammar for polymers . . . 4
- 3.2 *BcForms* grammar for complexes . . . 6

4 Formal descriptions of the grammars for polymers and complexes **6**
- 4.1 *BpForms* grammar for polymers . . . 6
- 4.2 *BcForms* grammar for complexes . . . 9

5 Coordinate system **10**

6 Construction of the alphabets of DNA, RNA, and protein residues **10**

7 Construction of the ontology of crosslinks **11**

8 Managing community contributions to *BpForms* and *BcForms* **11**

9 Semantic verification of polymers and complexes **11**

10 Integration with omics and systems and synthetic biology formats **12**
- 10.1 FASTA format for DNA, RNA, and protein sequences . . . 12
- 10.2 BioPAX format for pathways . . . 12
- 10.3 CellML and SBML formats for kinetic models . . . 12
- 10.4 Synthetic Biology Open Language (SBOL) format for genetic designs . . . 13

11 User interfaces **15**
- 11.1 Web applications . . . 15
- 11.2 REST APIs . . . 16
- 11.3 Command line programs . . . 16
- 11.4 Python libraries . . . 17

12 Implementation **17**

13 Comparisons with other formats and resources **19**
- 13.1 Comparison of the *BpForms* grammar with other formats . . . 19
- 13.2 Comparison of the *BpForms* alphabets with other resources . . . 21
- 13.3 Comparison of the *BpForms* crosslink ontology with other resources . . . 23
- 13.4 Comparison of the *BcForms* grammar with other formats . . . 23

14 Additional information for case studies in the main text **24**

15 Glossary **25**

16 Acronyms **25**

1. Introduction

This document provides additional information for several topics in Lang et al., 2020. Most of the information below is also available from the online user and developer tutorials and documentations at the locations listed below. In particular, the contents of the boxes are also available from the hyperlinks in their captions.

- Web applications and documentation of the grammars:
  https://bpforms.org
  https://bcforms.org
- Installation instructions for the command line programs and Python libraries:
  https://docs.karrlab.org/bpforms/installation.html
  https://docs.karrlab.org/bcforms/installation.html
- Tutorials for the grammars and Python libraries and interactive versions of the boxes below:
  https://sandbox.karrlab.org
- Documentation for the REST APIs:
  https://bpforms.org/api
  https://bpforms.org/api
- Documentation for the Python libraries:
  https://docs.karrlab.org/bpforms
  https://docs.karrlab.org/bcforms

2. Features of the *BpForms-BcForms* toolkit

The toolkit has the following features:

- **Concrete:** To help researchers communicate and integrate data about macromolecules, the grammars can capture the primary structures of macromolecules, including non-canonical (NC) residues, caps, crosslinks, and nicks.
- **Abstract:** To facilitate network research, the toolkit uses alphabets of residues and an ontology of crosslinks to abstract the structures of polymers and complexes.
- **Extensible:** To capture any polymer or complex, users can define residues and crosslinks inline or define custom alphabets and ontologies.
- **Structured coordinates:** To compose residues and crosslinks into polymers and complexes, each subunit, residue, and atom has a unique coordinate relative to its parent.
- **Context-free:** To help integrate information about the processes which synthesize and modify macromolecules, the grammars capture the structures of macromolecules separately from the processes which generate them.
- **User-friendly:** To ensure the toolkit is easy to use, the grammars are human-readable, and the toolkit includes web applications and command-line programs.
- **Machine-readable:** The grammars are machine-readable to enable analyses of macromolecules.

- **Composable:** To facilitate network research, we have developed protocols for composing the grammars with formats such as BioPAX, CellML, SBML, and SBOL.
- **Backward-compatible:** *BpForms* is backward compatible with the IUPAC/IUBMB format to maximize compatibility with existing formats, software, and knowledge.

3. Overview of the grammars for polymers and complexes

Below is an overview of the *BpForms* grammar for polymers and the *BcForms* grammar for complexes. The same information is also available from the online user guides for *BpForms* and *BcForms* at https://bpforms.org and https://bcforms.org.

3.1. *BpForms* grammar for polymers

The *BpForms* grammar captures polymers as a sequence of residues and nicks, optionally followed by a set of crosslinks and an optional indicator of circularity. The grammar captures sequences of residues using a similar syntax to the IUPAC/IUBMB format (often also known as the FASTA format). Residues that have single-character codes are indicated by their codes; residues that have multiple-character codes are indicated by enclosing their codes in curly brackets. In addition, users can define residues inline by enclosing several pairs of attributes and values in square brackets. For example, in the DNA alphabet, `Ga{dI}TCA` describes a 6-mer which contains 6-methyladenine (a) at the second residue and deoxyinosine at the third residue. In the protein alphabet, `RCAC | x-link: [type: "disulfide" | l: 2 | r: 4]` describes a 4-mer which contains a disulfide bond between the cysteines at the second and fourth positions. In the RNA alphabet, `AUUCG | circular` describes a circular 5-mer.

Here, we summarize the grammar. Box 1 and https://bpforms.org contain several examples of polymers encoded in the grammar. Section 4.1 contains a formal description of the grammar.

Residue sequence. Residues that belong to alphabets – collections of codes that denote specific residues (see main text) – can be indicated by their codes. Residues that have single-character codes can be indicated by their codes. For example, in the DNA alphabet, `A` describes deoxyadenosine monophosphate. Residues that have multiple-character codes can be indicated by enclosing their codes in brackets. For example, in the DNA alphabet `{m2C}` describes 2-O-methylcytidine monophosphate.

Residues that are not in the alphabets can be described in three ways: users can submit pull requests to add residues to the public alphabets, users can define their own alphabets, or users can define residues inline within descriptions of polymers.

User-defined residues. Residues can be defined inline as a pipe-separated set of pairs of attributes and values enclosed in square brackets. For example, `[name: "m6G" | ...]` describes 6-O-methylguanosine monophosphate (m^6G). The `structure` attribute can capture the molecular structure of the residue in SMILES format [1]. For example, `structure: "COc1nc(N)nc2c1ncn2[C@H]1C[C@@H]([C@H](O1)COP(=O)([-])[O-])O"` represents the structure of m^6G. Optionally, the `l-bond-atom` and `r-bond-atom` attributes can capture the atoms which can form bonds with preceding (l, left) and following (r, right) residues, and the `l-displaced-atom` and `r-displaced-atom` attributes can capture the atoms which are displaced by the formation of these bonds. The values of these attributes indicate the element, coordinate, and change in the formal charge of each atom upon bonding an adjacent residue. For example, `l-bond-atom: P23` indicates the phosphorous of m^6G that forms bonds with preceding residues and `l-displaced-`

`atom: O26-1` indicates the oxygen which is displaced by the formation of these bonds.

Several optional attributes can capture metadata about residues. The `id`, `name`, and `synonym` attributes can capture labels. The `identifier` attribute can capture references to equivalent entries in databases such as ChEBI [2]. For example, `identifier: "6-O-methylguanine" @ "dnamod"` indicates a reference to an entry in DNAmod. The `base-monomer` attribute can indicate how residues are synthesized from other residues. For example, `base-monomer: "G"` indicates that m^{6}G is derived from guanosine (G). The `comments` attribute can capture additional information about residues.

Crosslinks. *BpForms* represents each crosslink between two residues as (a) a pair of the atoms which form a bond between the residues and (b) a set of the atoms which are displaced by the formation of the bond. Crosslinks which belong to the ontology of crosslinks can be described by the `x-link` keyword followed by a pipe-separated list of pairs of attributes and values enclosed in square brackets. The `type` attribute indicates the type of the crosslink. The value of this attribute must refer to an entry in the crosslinks ontology. The `l` and `r` attributes indicate the coordinates of the residues involved in the crosslink. For example, `x-link: [type: "disulfide" | l: 2 | r: 5]` indicates a disulfide bond between cysteines at the second and fifth residues.

Users can also define crosslinks by submitting pull requests to add crosslinks to the public ontology, defining their own ontology, or defining crosslinks inline within descriptions of polymers.

User-defined crosslinks. Crosslinks can be defined inline as a pipe-separated set of pairs of attributes and values enclosed in square brackets. Similar to user-defined residues, the `l-bond-atom` and `r-bond-atom` attributes describe the atoms which form covalent bonds and the `l-displaced-atom` and `r-displaced-atom` attributes describe the atoms which are displaced by the formation of these bonds. For example, `l-bond-atom: 2O11-1 | r-bond-atom: 7P20` indicates that the crosslink involves a covalent bond between the oxygen at the eleventh position of the second residue and the phosphorous at the twentieth position of the seventh residue, and that the formation of the crosslink decreases the formal charge of the oxygen by one electron. The `order` attribute can capture the order (single, double, triple, or aromatic) of the bond. The `stereo` attribute can capture the stereochemistry (wedge, hash, up, or down) of the bond. The `comments` attribute can capture additional textual information about the crosslink.

Nicks. Nicks can be used to describe the absence of an inter-residue bond between successive residues, such as a strand break in DNA, which separates a polymer into two fragments. Nicks are intended to be used in conjunction with crosslinks which link the fragments despite the absence of a continuous chain of inter-residue bonds.

Macromolecules that contain fragments separated nicks and joined by crosslinks can also be described as complexes with *BcForms*. For some use cases, nicks offer a more natural way to describe such macromolecules.

Nicks can be indicated by inserting a colon between the residues involved in the nick. For example, `ACG:T` describes a nick between the third and fourth residues of a DNA molecule and `AC:DE` describes a nick between the second and third residues of a peptide.

Linear or circular topology. Optionally, the `circular` attribute can describe a bond between the left bonding site of the first residue and the right bonding site of the last residue. For example, `CTAC | circular` describes a circular DNA tetramer.

Missing knowledge. User-defined residues can also capture four types of uncertainty about polymers. The `delta-mass` and `delta-charge` attributes can describe mass and charge which have been observed, but which cannot be interpreted as a specific molecular structure. For example, `[identifier: "ARG" @ "pdb-cc" | structure: "OC(=O)[C@H](CCCNC(=[NH2+])N)[NH3+]" | r-bond-atom: C2 | l-bond-atom: N15-1 | r-displaced-atom: O1 | r-displaced-atom: H1 | l-displaced-atom: H15+1 | l-displaced-atom: H15 | delta-mass: 17 | delta-charge: 0]` indicates a residue whose mass is 17 Da greater than that of arginine, but whose exact structure is not known. The `position` attribute can capture uncertainty about the location and biosynthesis of an NC residue. For example, `AC[base-monomer: "Y" | identifier: "MOD:00696" @ "mod" | position: 3-5 [S, T, Y]]ST` indicates a peptide that contains a phosphorylated serine, threonine, or tyrosine between positions five and ten.

3.2. *BcForms* grammar for complexes

BcForms describes complexes using a grammar that is similar to a linear mathematical expression. For example, `CHAF1A + SUMO1 | x-link: [ ...]` describes a crosslinked heterodimer of chromatin assembly factor 1 subunit A (CHAF1A) and small ubiquitin-related modifier 1 (SUMO1).

Here, we summarize the grammar. Box 2 and https://bcforms.org contain examples of complexes encoded in the grammar. Section 4.2 contains a formal description of the grammar.

Subunit composition. The subunits involved in complexes and their stoichiometries can be described as a linear expression. For example, `2 * HBA1 + 2 * HBB` describes hemoglobin HbA, a heterotetramer composed of two subunits of HBA1 (UniProt: P69905) and two subunits of HBB (UniProt: P68871). Subunits which are DNA, RNA, or protein polymers can be represented using *BpForms*; subunits which are small molecules, such as vitamins, can be represented using SMILES.

Intersubunit crosslinks. *BcForms* captures crosslinks similar to *BpForms*. Crosslinks which belong to the ontology can be described using the coordinates of the residues involved in the crosslink. For example, `x-link: [type: "disulfide" | l: P83658(1)-7 | r: P83658(2)-12 | ...]` describes a disulfide bond between the seventh and twelfth cysteines of two subunits of disintegrin schistatin (UniProt: P83658). The `l` and `r` attributes describe the subunit type, subunit coordinate, and residue coordinate of the atoms involved in the crosslink. Users can also define crosslinks inline similarly to *BpForms*. For example, `x-link: [l-bond-atom: P83658(1)-7S11 | r-bond-atom: P83658(2)-12S11 | ...]` describes the same disulfide bond between the seventh and twelfth cysteines of disintegrin schistatin.

Complexes can have zero, one, or more crosslinks. Each crosslink can involve the formation of one or more covalent bonds and the displacement of zero or more atoms.

4. Formal descriptions of the grammars for polymers and complexes

The *BpForms* and *BcForms* grammars are defined in Extended Backus-Naur Form (EBNF) [3] using Lark [4]. The grammars are available at https://github.com/KarrLab/bpforms and https://github.com/KarrLab/bcforms. Below are descriptions of the grammars in Backus-Naur Form (BNF).

4.1. *BpForms* grammar for polymers

BpForm

$$\langle\text{bpform}\rangle \models \langle\text{seq}\rangle\ \langle\text{x-links}\rangle\ \langle\text{circularity}\rangle$$

Sequence of residues and nicks		
⟨seq⟩	⊨	⟨residue⟩ | ⟨residue⟩ ⟨seq⟩ | *nick* ⟨residue⟩ ⟨seq⟩
⟨residue⟩	⊨	⟨single-code-residue⟩ | ⟨delimited-multi-code-residue⟩ | ⟨user-residue⟩
Alphabet-defined residues		
⟨single-code-residue⟩	⊨	⟨code⟩
⟨delimited-multi-code-residue⟩	⊨	`{` ⟨multi-code⟩ `}`
⟨code⟩	⊨	*non-whitespace character*
⟨multi-code⟩	⊨	⟨code⟩ | ⟨code⟩ ⟨multi-code⟩
User-defined residues		
⟨user-residue⟩	⊨	`[` ⟨attrs⟩ `]`
⟨attrs⟩	⊨	⟨attr⟩ | ⟨attr⟩ `'|'` ⟨attrs⟩ | λ
⟨attr⟩	⊨	⟨id⟩ | ⟨name⟩ | ⟨synonym⟩ | ⟨identifier⟩ | ⟨structure⟩ | ⟨atom⟩ | ⟨base⟩ | ⟨delta-mass⟩ | ⟨delta-charge⟩ | ⟨position⟩ | ⟨comments⟩
⟨id⟩	⊨	`id` : " ⟨escaped-string⟩ "
⟨name⟩	⊨	`name` : " ⟨escaped-string⟩ "
⟨synonym⟩	⊨	`synonym` : " ⟨escaped-string⟩ "
⟨identifier⟩	⊨	`identifier` : " ⟨identifier-ns⟩ " `@` " ⟨identifier-id⟩ "
⟨identifier-ns⟩	⊨	⟨escaped-string⟩
⟨identifier-id⟩	⊨	⟨escaped-string⟩
⟨structure⟩	⊨	`structure` : " ⟨string⟩ "
⟨atom⟩	⊨	⟨atom-type⟩ : ⟨atom-element⟩ ⟨atom-index⟩ ⟨atom-charge⟩
⟨base⟩	⊨	`base-monomer` : " ⟨multi-code⟩ "
⟨delta-mass⟩	⊨	`delta-mass` : ⟨number⟩
⟨delta-charge⟩	⊨	`delta-charge` : ⟨integer⟩
⟨position⟩	⊨	`position` : ⟨position-start⟩ - ⟨position-end⟩ ⟨position-residues⟩
⟨position-start⟩	⊨	⟨positive-integer⟩
⟨position-end⟩	⊨	⟨positive-integer⟩
⟨position-residues⟩	⊨	`[` ⟨position-residue-codes⟩ `]` | λ
⟨position-residue-codes⟩	⊨	⟨multi-code⟩ | ⟨multi-code⟩ `'|'` ⟨position-residue-codes⟩
⟨comments⟩	⊨	`comments` : " ⟨escaped-string⟩ "
Crosslinks		
⟨x-links⟩	⊨	⟨x-link⟩ | ⟨x-link⟩ ⟨x-links⟩ | λ
⟨x-link⟩	⊨	`'|'` `x-link` : `[` ⟨x-link-attrs⟩ `]`
⟨x-link-attrs⟩	⊨	⟨onto-x-link-attrs⟩ | ⟨user-x-link-attrs⟩

⟨onto-x-link-attrs⟩	⊨	⟨onto-x-link-attr⟩ | ⟨onto-x-link-attr⟩ `'|'` ⟨onto-x-link-attrs⟩
⟨onto-x-link-attr⟩	⊨	⟨onto-x-link-type⟩ | ⟨onto-x-link-l-monomer⟩ | ⟨onto-x-link-r-monomer⟩
⟨onto-x-link-type⟩	⊨	`type : "` ⟨non-whitespace-characters⟩ `"`
⟨onto-x-link-l-monomer⟩	⊨	`l :` ⟨positive-integer⟩
⟨onto-x-link-r-monomer⟩	⊨	`r :` ⟨positive-integer⟩
User-defined crosslinks		
⟨user-x-link-attrs⟩	⊨	⟨user-x-link-attr⟩ | ⟨user-x-link-attr⟩ `'|'` ⟨user-x-link-attrs⟩ | λ
⟨user-x-link-attr⟩	⊨	⟨user-x-link-atom⟩ | ⟨user-x-link-order-attr⟩ | ⟨user-x-link-stereo-attr⟩ | ⟨user-x-link-comments-attr⟩
⟨user-x-link-atom⟩	⊨	⟨atom-type⟩ ⟨atom-residue⟩ ⟨atom-element⟩ ⟨atom-index⟩ ⟨atom-charge⟩
⟨user-x-link-order-attr⟩	⊨	`order : "`⟨user-x-link-order⟩ `"`
⟨user-x-link-order⟩	⊨	`single` | `double` | `triple` | `aromatic`
⟨user-x-link-stereo-attr⟩	⊨	`stereo : "`⟨user-x-link-stereo⟩ `"`
⟨user-x-link-stereo⟩	⊨	`wedge` | `hash` | `up` | `down`
⟨user-x-link-comments-attr⟩	⊨	`commments : "` ⟨escaped-string⟩ `"`
nicks		
⟨nick⟩	⊨	`:`
Circularity		
⟨circularity⟩	⊨	`'|' circular` | λ
User-defined atoms		
⟨atom-type⟩	⊨	`l-bond-atom` | `l-displaced-atom` | `r-bond-atom` | `r-displaced-atom`
⟨atom-residue⟩	⊨	⟨positive-integer⟩
⟨atom-element⟩	⊨	`A...Z` | `A...Z a...z`
⟨atom-index⟩	⊨	⟨positive-integer⟩
⟨atom-charge⟩	⊨	⟨sign⟩ ⟨non-negative-integer⟩ | λ
⟨sign⟩	⊨	`+` | `-`
Primitives		
⟨string⟩	⊨	*string*
⟨escaped-string⟩	⊨	*quote escaped string*
⟨non-whitespace-characters⟩	⊨	*non-whitespace characters*
⟨integer⟩	⊨	*integer*

⟨positive-integer⟩ ⊨ *positive integer*
⟨non-negative-integer⟩ ⊨ *non-negative integer*

4.2. *BcForms* grammar for complexes

BcForm

⟨bcform⟩ ⊨ ⟨subunits⟩ ⟨x-links⟩

Subunits

⟨subunits⟩ ⊨ ⟨subunit⟩ | ⟨subunit⟩ + ⟨subunits⟩
⟨subunit⟩ ⊨ ⟨coefficient⟩ * ⟨id⟩ | ⟨id⟩
⟨id⟩ ⊨ ⟨non-white space characters⟩
⟨coefficient⟩ ⊨ ⟨positive integer⟩

Crosslinks

⟨x-links⟩ ⊨ ⟨x-link⟩ | ⟨x-link⟩ ⟨x-links⟩
⟨x-link⟩ ⊨ '|' `crosslink` : [⟨x-link-attrs⟩]
⟨x-link-attrs⟩ ⊨ ⟨onto-x-link-attrs⟩ | ⟨user-x-link-attrs⟩
⟨onto-x-link-attrs⟩ ⊨ ⟨onto-x-link-attr⟩ | ⟨onto-x-link-attr⟩ '|' ⟨onto-x-link-attrs⟩
⟨onto-x-link-attr⟩ ⊨ ⟨onto-x-link-type⟩ | ⟨onto-x-link-l-monomer⟩ | ⟨onto-x-link-r-monomer⟩
⟨onto-x-link-type⟩ ⊨ `type` : " ⟨onto-x-link-type-value⟩ "
⟨onto-x-link-type-value⟩ ⊨ ⟨non-whitespace characters⟩
⟨onto-x-link-l-monomer⟩ ⊨ `l` : ⟨atom-subunit-id⟩ (⟨atom-subunit-index⟩) - ⟨atom-monomer-index⟩
⟨onto-x-link-r-monomer⟩ ⊨ `r` : ⟨atom-subunit-id⟩ (⟨atom-subunit-index⟩) - ⟨atom-monomer-index⟩

User-defined crosslinks

⟨user-x-link-attrs⟩ ⊨ ⟨user-x-link-attr⟩ | ⟨user-x-link-attr⟩ '|' ⟨user-x-link-attrs⟩
⟨user-x-link-attr⟩ ⊨ ⟨user-x-link-atom⟩ | | ⟨user-x-link-order-attr⟩ | ⟨user-x-link-stereo-attr⟩ | ⟨user-x-link-comments-attr⟩
⟨user-x-link-order-attr⟩ ⊨ `order` : "⟨user-x-link-order⟩ "
⟨user-x-link-order⟩ ⊨ `single` | `double` | `triple` | `aromatic`
⟨user-x-link-stereo-attr⟩ ⊨ `stereo` : "⟨user-x-link-stereo⟩ "
⟨user-x-link-stereo⟩ ⊨ `wedge` | `hash` | `up` | `down`
⟨user-x-link-comments-attr⟩ ⊨ `commments` : " ⟨escaped-string⟩ "

User-defined atoms

⟨user-x-link-atom⟩ ⊨ ⟨atom-type⟩ `:` ⟨atom-subunit-id⟩ `(` ⟨atom-subunit-index⟩ `)` `-` ⟨atom-monomer-index⟩ ⟨atom-index⟩ ⟨atom-element⟩ ⟨atom-charge⟩

⟨atom-type⟩ ⊨ `l-bond-atom` | `r-bond-atom` | `l-displaced-atom` | `r-displaced-atom`

⟨atom-subunit-id⟩ ⊨ ⟨non-whitespace characters⟩

⟨atom-subunit-index⟩ ⊨ ⟨positive integer⟩

⟨atom-monomer-index⟩ ⊨ ⟨positive integer⟩

⟨atom-index⟩ ⊨ ⟨positive integer⟩

⟨atom-element⟩ ⊨ `A...Z` | `A...Z a...z`

⟨atom-charge⟩ ⊨ ⟨sign⟩ ⟨atom-charge-value⟩ | λ

⟨sign⟩ ⊨ `+` | `-`

⟨atom-charge-value⟩ ⊨ ⟨non-negative integer⟩

Primitives

⟨escaped-string⟩ ⊨ *quote escaped string*

⟨non-whitespace-characters⟩ ⊨ *non-whitespace characters*

⟨positive-integer⟩ ⊨ *positive integer*

⟨non-negative-integer⟩ ⊨ *non-negative integer*

5. Coordinate system

To facilitate descriptions of inter-residue bonds and crosslinks, each residue, and atom represented by *BpForms* has a unique coordinate (Figure 1). The coordinate of each residue is its position within the residue sequence of its parent polymer. The coordinate of each atom is a tuple of the coordinate of its parent residue and its position within the canonical SMILES ordering of the atoms in its parent residue before incorporation into polymers.

Each subunit, residue, and atom represented by *BcForms* also has a unique coordinate (Figure 1). The coordinates of repeated subunits range from one to the stoichiometry of the subunit. The coordinate of each residue is a two-tuple of the coordinate of its parent subunit and its position within the residue sequence of its parent subunit. The coordinate of each atom is a three-tuple of the coordinate of its parent subunit, the position of its parent residue within the residue sequence of its parent polymer, and its position within the canonical SMILES ordering of its parent residue.

6. Construction of the alphabets of DNA, RNA, and protein residues

To support a broad range of research, we developed the alphabets of DNA, RNA, and protein residues by merging residues from multiple databases. We developed the DNA alphabet by combining the deoxyribose nucleotide monophosphates and 3' and 5' DNA ends from the PDB Chemical Component Dictionary (PDB CCD) [5] with the verified DNA nucleobases from DNAmod [6] and the deoxyribose nucleosides from REPAIRtoire [7] that had concrete structures. We developed the RNA alphabet by combining the ribose nucleotide monophosphates and 3' and 5' RNA ends

from the PDB CCD with the ribose nucleosides from MODOMICS [8] and the RNA Modification Database [9] that had concrete structures. We developed the protein alphabet by merging residues and ends from the PDB CCD and RESID [10].

First, we downloaded, scraped, and manually extracted residues from DNAmod, MODOMICS, the PDB CCD, REPAIRtoire, RESID, the RNA Modification Database. Second, we parsed each database into a list of residues. Third, we rejected residues with incompletely defined structures, as well as inconsistent residues such as nucleotides from DNAmod. Fourth, we normalized the DNA and RNA residues to nucleotide monophosphates and normalized the protein residues to amino acids. For example, we transformed the DNAmod entries to nucleotides by adding deoxyribose monophosphate to each nucleobase. Fifth, we merged the repeated residues. This included residues that had the same molecular structure, that the upstream sources annotated were equivalent, or that had similar names. Lastly, we identified the atom indices of the left/preceding and right/following bonding sites in each residue. For DNA and RNA, the left and right bonding sites comprised the phosphorus atom in the phosphate group bonded to the 5' carbon and the oxygen atom bonded to the 3' carbon, respectively. For protein residues, these comprised the nitrogen atom in the amino group and the acidic oxygen atom in the carboxyl group.

We automated the alphabet construction process by writing scripts to build each alphabet. Going forward, this will enable us to easily incorporate updates to the upstream databases into the alphabets.

7. Construction of the ontology of crosslinks

We developed the ontology of crosslinks based on entries in RESID which represent crosslinked dipeptides. First, we searched RESID for entries that represent crosslinked dipeptides. Second, we identified the individual residues which participate in each dimer. Third, we used ChemAxon Marvin [11] to identify the atoms involved in each crosslink. Next, we used Open Babel [12] to determine the indices of these atoms in the canonical SMILES ordering of the atoms. Finally, we manually assigned an id and name to each crosslink.

Currently, the ontology contains crosslinks between protein residues. Going forward, we hope to work with the community to curate additional crosslinks, including DNA-DNA, RNA-RNA, DNA-protein, and RNA-protein crosslinks.

8. Managing community contributions to *BpForms* and *BcForms*

We welcome contributions to *BpForms* and *BcForms*, including to the software, grammars, alphabets of residues, and ontology of crosslinks. We encourage potential contributors to initiate discussion by email. For now, we encourage the community to use GitHub pull requests to contribute to *BpForms* and *BcForms*. More information is available at http://docs.karrlab.org/bpforms.

9. Semantic verification of polymers and complexes

To help quality control information about macromolecules, *BpForms* and *BcForms* include methods for verifying the semantic correctness of polymers and complexes. *BpForms* checks that each residue has a defined structure, each atom that bonds an adjacent residue has a defined element and position which is consistent with the structure of its parent residue, and each pair of consecutive residues

can form a bond. *BpForms* also checks that the element and position of each atom in each crosslink are consistent with the structure of its parent residue. For example, *BpForms* can identify invalid proteins that contain consecutive residues that cannot bond because the first residue lacks a carboxyl terminus or the second residue lacks an amino terminus.

BcForms checks that each subunit is semantically concrete and that the element and position of each atom in each crosslink are consistent with the structure of its parent residue.

10. Integration with omics and systems and synthetic biology formats

This section illustrates how to use *BpForms* and *BcForms* to annotate the semantic meaning of the species involved in genomics datasets, pathways, models, and genetic designs. The examples in the boxes below are also available from GitHub at the hyperlinks in the captions.

10.1. FASTA format for DNA, RNA, and protein sequences

Box S1 illustrates how *BpForms* can be integrated with the FASTA format [13] to describe multiple NC DNA, RNA, or proteins within a single file. The *BpForms* Python library includes methods for encoding and decoding *BpForms* into and out of FASTA documents. *BpForms*-encoded FASTA documents can also be read and written by standard FASTA tools such as Biopython [14]. However, these tools cannot interpret the semantic meaning of molecules encoded in *BpForms*.

10.2. BioPAX format for pathways

BioPAX [15] is a format for describing biochemical pathways such as signaling. Box S2 illustrates how *BpForms* can be integrated with BioPAX to describe the polymers that participate in pathways. Users who need to describe residues and crosslinks which are not part of the public *BpForms* ontologies can either describe the residues and crosslinks inline inside descriptions of polymers, build custom alphabets of residues and a custom ontology of crosslinks and bundle these documents with BioPAX documents into COMBINE archives [16], or submit GitHub pull requests to add residues and crosslinks to the public alphabets and ontology. By helping BioPAX describe the polymers involved in pathways, *BpForms* can make pathways easier to understand and combine into comprehensive maps of cells. Unfortunately, there is no straightforward way to integrate *BcForms* with BioPAX because BioPAX includes a competing data model for complexes which is not extensible.

10.3. CellML and SBML formats for kinetic models

CellML [17] and the Systems Biology Markup Language (SBML) [18] are formats for describing kinetic models. Both formats have limited capabilities to describe the semantic meaning of model elements which represent macromolecules because both formats encourage users to annotate the

```
> yp | phosphorylated MEK | Q02750 | pS218
MPKKKPTPIQLNPAPDGSAVNGTSSAETNLEALQKKLEELELDEQQRKRLEAFLTQKQKVGELKDDDFEKISELGAGNGGVVFKV
SHKPSGLVMARKLIHLEIKPAIRNQIIRELQVLHECNSPYIVGFYGAFYSDGEISICMEHMDGGSLDQVLKKAGRIPEQILGKVS
IAVIKGLTYLREKHKIMHRDVKPSNILVNSRGEIKLCDFGVSGQLID{AA0037}MANSFVGTRSYMSPERLQGTHYSVQSDIWS
MGLSLVEMAVGRYPIPPPDAKELELMFGCQVEGDAAETPPRPRTPGRPLSSYGMDSRPPMAIFELLDYIVNEPPPKLPSGVFSLE
FQDFVNKCLIKNPAERADLKQLMVHAFIKRSDAEEVDFAGWLCSTIGLNQPSTPTHAAGV
```

Box S1. ***BpForms* can be integrated with the FASTA format to describe multiple polymers within a single document.** For example, a FASTA document can contain a *BpForms*-encoded description of monophosphorylated MAPK (UniProt: Q02750). The full example FASTA document is available at GitHub.

```
...
<bp:DNA>
   <bp:entityReference>
      <bp:DNAReference>
         <bp:sequence
            rdf:datatype="http://www.w3.org/2001/XMLSchema#string"
            rdf:about="http://edamontology.org/format_3909#dna">
            ...
            TGATTTGCCGTGGCGAGAAAATGTCG{a}TCGCCATTATGGCCGGCGTATTA
            GAAGCGCGCGGTCACAACGTTACTGTTATCG{a}TCCGGTCGAAAAACTGCT
            ...
         </bp:sequence>
      </bp:DNAReference>
   </bp:entityReference>
</bp:DNA>
...
```

Box S2. ***BpForms* can help BioPAX documents describe the polymers involved in pathways.** For example, *BpForms* can help BioPAX capture the DNA methylation (orange) that helps *Escherichia coli* detect and degrade foreign DNA. Positions 701 to 800 of *E. coli*'s genome are shown. The full example BioPAX document is available at GitHub.

meaning of model elements by referencing entities in databases such as UniProt [19] which do not represent every possible form of every macromolecule. For example, CellML and SBML have limited capabilities to capture the differences among the monophosphorylated states of MAPK because UniProt only contains one entry per protein. Specifically, UniProt does not support distinct entries for each form of each protein. Rather, all of the forms of a protein are encompassed by a single UniProt entry.

Boxes S3 and S4 illustrate how *BpForms* and *BcForms* can be integrated with CellML and SBML to describe the macromolecules represented by models. Users who need to describe residues and crosslinks which are not part of the public *BpForms* ontologies can either (a) describe the residues and crosslinks inline inside descriptions of polymers, (b) build custom alphabets of residues and a custom ontology of crosslinks and bundle these documents with CellML and SBML documents into COMBINE archives [16], or (c) submit GitHub pull requests to add residues and crosslinks to the public ontologies.

By describing the semantic meaning of model elements, *BpForms* and *BcForms* can make models easier to understand, compare, extend, and combine into more comprehensive models such as whole-cell (WC) models [21, 22].

10.4. Synthetic Biology Open Language (SBOL) format for genetic designs

SBOL [24] is a format for describing genetic designs for synthetic organisms. Box S5 illustrates how *BpForms* can be integrated with SBOL to concretely describe the DNA, RNA, and protein parts of genetic designs. Users who need to describe residues and crosslinks which are not part of the public *BpForms* ontologies can either (a) describe the residues and crosslinks inline inside descriptions of polymers, (b) build custom alphabets of residues and a custom ontology of crosslinks and bundle these documents with SBOL documents into COMBINE archives [16], or (C) submit GitHub pull requests to add residues and crosslinks to the public alphabets and ontology. In SBOL PEP 033 [25], we formally proposed this integration between *BpForms* and SBOL to the SBOL community. Unfortunately, there is no straightforward way to integrate *BcForms* with SBOL because SBOL includes a competing data model for complexes which is not extensible. Instead, we encourage the SBOL community to expand SBOL to capture stoichiometric and crosslink information about

```
...

<species metaid="cdc2k">
   <annotation>
      <rdf:RDF
         xmlns:rdf="http://www.w3.org/1999/02/22-rdf-syntax-ns#">
         <rdf:Description rdf:about="#cdc2k">
            <bpforms:ProteinForm xmlns:bpforms="https://bpforms.org">
               MENYQKVEKIGEG{AA0038}{AA0039}GVVYKARHKLSGRIVAMKKIRLEDESEGVPSTAIREISLLKE
               VNDENNRSNCVRLLDILHAESKLYLVFEFLDMDLKKYMDRISETGATSLDPRLVQKFTYQLVNGVNFCHSR
               RIIHRDLKPQNLLIDKEGNLKLADFGLARSFGVPLRNY{AA0038}HEIVTLWYRAPEVLLGSRHYSTGVD
               IWSVGCIFAEMIRRSPLFPGDSEIDEIFKIFQVLGTPNEEVWPGVTLLQDYKSTFPRWKRMDLHKVVPNGE
               EDAIELLSAMLVYDPAHRISAKRALQQNYLRDFH
            </bpforms:ProteinForm>
         </rdf:Description>
      </rdf:RDF>
   </annotation>
</species>

<species metaid="YP">
   <annotation>
      <rdf:RDF xmlns:rdf="http://www.w3.org/1999/02/22-rdf-syntax-ns#">
         <rdf:Description rdf:about="#YP">
            <bpforms:ProteinForm xmlns:bpforms="https://bpforms.org">
               MTTRRLTRQHLLANTLGNNDENHPSNHIARAK{AA0037}{AA0037}LH{AA0037}{AA0037}EN{AA
               0037}LVNGKKATVSSTNVPKKRHALDDV{AA0037}NFHNKEGVPLASKNTNVRHTTASVSTRRALEEKS
               IIPATDDEPA{AA0037}KKRRQPSVFNSSVPSLPQHLSTKSHSVSTHGVDAFHKDQATIPKKLKKDVDER
               VVSKDIPKLHRDSVESPESQDWDDLDAEDWADPLMVSEYVVDIFEYLNELEIETMPSPTYMDRQKELAWKM
               RGILTDWLIEVHSRFRLLPETLFLAVNIIDRFLSLRVCSLNKLQLVGIAALFIASKYEEVMCPSVQNFVYM
               ADGGYDEEEILQAERYILRVLEFNLAYPNPMNFLRRISKADFYDIQTRTVAKYLVEIGLLDHKLLPYPPSQ
               QCAAAMYLAREMLGRGPWNRNLVHYSGYEEYQLISVVKKMINYLQKPVQHEAFFKKYASKKFMKASLFVRD
               WIKKNSIPLGDDADEDYTFHKQKRIQHDMKDEEW
            </bpforms:ProteinForm>
         </rdf:Description>
      </rdf:RDF>
   </annotation>
</species>

<species metaid="pM" name="p-cyclin_cdc2-p">
   <annotation>
      <rdf:RDF xmlns:rdf="http://www.w3.org/1999/02/22-rdf-syntax-ns#">
         <rdf:Description rdf:about="#YP">
            <bcforms:BcForm xmlns:bcforms="https://bcforms.org">
               YP + cdc2k
            </bcforms:BcForm>
         </rdf:Description>
      </rdf:RDF>
   </annotation>
</species>

...
```

Box S3. ***BpForms* can help SBML describe the semantic meaning of the macromolecules represented by models.** For example, *BpForms* can help SBML describe that the cdc2k species of the Tyson cell cycle model [20] represents a tri-phosphorylated form of cyclin dependent kinase 1 (UniProt: P04551) and that the YP species represents a 7-phosphorylated form of G2/mitotic-specific cyclin cdc13 (UniProt: P10815. The full example SBML document is available at GitHub.

```
...
<component cmeta:id="ypp" name="ypp">
   <rdf:RDF xmlns:rdf="http://www.w3.org/1999/02/22-rdf-syntax-ns#">
      <rdf:Description rdf:about="#ypp">
         <bpforms:ProteinForm xmlns:bpforms="https://bpforms.org">
            MPKKKPTPIQLNPAPDGSAVNGTSSAETNLEALQKKLEELELDEQQRKRLEAFLTQKQKVGELKDDDFEKISEL
            GAGNGGVVFKVSHKPSGLVMARKLIHLEIKPAIRNQIIRELQVLHECNSPYIVGFYGAFYSDGEISICMEHMDG
            GSLDQVLKKAGRIPEQILGKVSIAVIKGLTYLREKHKIMHRDVKPSNILVNSRGEIKLCDFGVSGQLID{AA00
            37}MAN{AA0037}FVGTRSYMSPERLQGTHYSVQSDIWSMGLSLVEMAVGRYPIPPPDAKELELMFGCQVEGD
            AAETPPRPRTPGRPLSSYGMDSRPPMAIFELLDYIVNEPPPKLPSGVFSLEFQDFVNKCLIKNPAERADLKQLM
            VHAFIKRSDAEEVDFAGWLCSTIGLNQPSTPTHAAGV
         </bpforms:ProteinForm>
      </rdf:Description>
   </rdf:RDF>
</component>
...
```

Box S4. ***BpForms*** **can help CellML describe the semantic meaning of components which represent macromolecules.** For example, *BpForms* can help CellML describe that the ypp variable in the Wang MAPK cascade model [23] represents a biphosphorylated form of MEK (UniProt: Q0275). The full example CellML document is available at GitHub.

complexes.

By helping capture the structures of parts, *BpForms* can help bioengineers identify the biosynthetic dependencies of parts and, in turn, identify constraints on transforming parts into alternate hosts. For example, *BpForms* can help bioengineers identify post-translational modification enzymes that must be co-transformed with parts to synthesize modifications that are essential to the functions of parts.

11. User interfaces

This section describes how to use the *BpForms* and *BcForms* user interfaces. The tutorials in the boxes below, as well as more detailed tutorials, are also available from https://docs.karrlab.org/bpforms, https://docs.karrlab.org/bcforms, and https://sandbox.karrlab.org.

11.1. Web applications

The *BpForms* and *BcForms* web applications are available at https://bpforms.org and https://bcforms.org. Both applications provide simple web applications for validating and calculating prop-

```
...

<sbol:Sequence>
   <sbol:elements>
      GGGCCUGUAGCUCAGC{8U}GG{8U}{8U}AGAGCGCACGCCUGAU{62A}AGCGUGAG{7G}UCGAUGG{5U}{9U}C
      GAGUCCAUUCAGGCCCACCA
   </sbol:elements>
   <sbol:encoding rdf:resource="http://edamontology.org/format_3909#rna"/>
</sbol:Sequence>

...
```

Box S5. ***BpForms*** **can help SBOL describe DNA, RNA, and protein parts.** For example, *BpForms* can help SBOL describe the post-transcriptional modifications required for *Bacillus subtilis* $tRNA^{Ile}$ 69 (KEGG: BSU_tRNA_69, SynBioHub: BO_28687). The full example SBOL document is available at GitHub.

erties of polymers and complexes.

11.2. REST APIs

The *BpForms* and *BcForms* REST APIs are available at https://bpforms.org/api and https://bcforms.org/api. OpenAPI specifications and documentation for the APIs are available at the same URLs. The documentation includes simple web forms for querying the APIs.

11.3. Command line programs

The *BpForms* and *BcForms* command line programs are available from PyPI [26]. Boxes S6 and S7 illustrate how to install and use the programs.

```
# Display help
bpforms --help

# Validate descriptions polymers
bpforms validate dna 'ACGT | circular'
» Form is valid

bpforms validate protein 'CRATUG'
» Form is valid

# Calculate properties of polymers
bpforms get-properties dna 'ACGT | circular'
» Length: 4
» Structure: O(C1CC(....
» Formula: C39H46N15O25P4
» Molecular weight: 1248.772047992
» Charge: -5

bpforms get-properties protein 'CRATUG'
» Length: 6
» Structure: C(=O)([C@@H](...
» Formula: C21H41N9O8SSe
» Molecular weight: 658.645
» Charge: 2
```

Box S6. Tutorial for the *BpForms* command line program. A more detailed version of the tutorial is also available at http://docs.karrlab.org.

```
# Display help
bcforms --help

# Validate descriptions of complexes
bcforms validate '2 * a + 3 * b'
» Form is valid

# Calculate properties of complexes
bcforms get-formula '2 * a + 3 * b' '{a: CHO, b: C2H2O2}'
» C8H8O8
bcforms get-charge '2 * a + 3 * b' '{a: 1, b: 2}'
» 8
```

Box S7. Tutorial for the *BcForms* command line program. A more detailed version of the tutorial is also available at http://docs.karrlab.org.

```
# Import library
import bpforms

# Create polymers from their string representations
dna_1 = bpforms.DnaForm().from_str('ACGT | circular')
rna_1 = bpforms.RnaForm().from_str('C{01A}GU')
prot_1 = bpforms.ProteinForm().from_str('CVYT{U}C | x-link:  [type:  "disulfide"'
                                        ' | l:  1 | r:  6]')

# Create the same polymers programmatically
dna_2 = bpforms.DnaForm()
for residue in ['A', 'C', 'G', 'T']:
   dna_2.seq.append(bpforms.dna_alphabet.monomers[residue])
dna_2.circular = True

rna_2 = bpforms.RnaForm()
for residue in ['C', '01A', 'G', 'U']:
   rna_2.seq.append(bpforms.rna_alphabet.monomers[residue])

prot_2 = bpforms.ProteinForm()
for residue in ['C', 'V', 'Y', 'T', 'U', 'C']:
   prot_2.seq.append(bpforms.protein_alphabet.monomers[residue])
prot_2.crosslinks.add(bpforms.OntoBond(
                       type=bpforms.xlink.crosslinks_onto['disulfide'],
                       l_monomer=1, r_monomer=6))

# Get properties of polymers
dna_1.circular »True
rna_1.seq[1] »<bpforms.core.Monomer at 0x7f89db9d9d68>
prot_1.crosslinks »{<bpforms.core.OntoBond at 0x7f0073054850>}

# Get the string representation of a polymer
str(dna_2) »ACGT | circular

# Check equality of polymers
dna_2.is_equal(dna_1) »True

# Calculate properties of a polymer
dna_1.get_structure()[0] »<openbabel.OBMol>
dna_1.export('smiles') »O1C2CC(OC2COP(=O)([O-])OC2CC(OC2COP(=O)...
str(dna_1.get_formula()) »C39H45N15O24P4
dna_1.get_charge() »-4

# Get the canonical sequence of a polymer
rna_1.get_canonical_seq() »CAGU
```

Box S8. Tutorial for the *BpForms* Python library. This tutorial and a more detailed tutorial are also available at https://sandbox.karrlab.org.

11.4. Python libraries

The *BpForms* and *BcForms* Python libraries are available from PyPI [26]. Boxes S8 and S9 provide brief tutorials for the libraries. More extensive, interactive tutorials are available as Jupyter notebooks at https://sandbox.karrlab.org.

12. Implementation

We implemented *BpForms* and *BcForms* with Python 3 [27] and several additional packages. We described the grammar in EBNF, and used Lark [4] to implement a parser for the grammar. We

```
# Import libraries
import bcforms
import bpforms

# Create complexes from their string representations
form_1 = bcforms.BcForm().from_str('2 * subunit_a + 3 * subunit_b')
form_1.set_subunit_attribute('subunit_a', 'structure',
   bpforms.ProteinForm().from_str('CAAAAAAAA'))
form_1.set_subunit_attribute('subunit_b', 'structure',
   bpforms.ProteinForm().from_str('AAAAAAAAC'))

form_2 = bcforms.BcForm().from_str(
   '2 * subunit_a'
   '| x-link: [type: disulfide | l: subunit_a(1)-1 | r: subunit_a(2)-1]')
form_2.set_subunit_attribute('subunit_a', 'structure',
   bpforms.ProteinForm().from_str('CAAAAAAAA'))

# Create complexes programmatically
form_1_b = bcforms.BcForm()
form_1_b.subunits.append(bcforms.core.Subunit('subunit_a', 2,
   bpforms.ProteinForm().from_str('CAAAAAAAA')))
form_1_b.subunits.append(bcforms.core.Subunit('subunit_b', 3,
   bpforms.ProteinForm().from_str('AAAAAAAAC')))

form_2_b = bcforms.BcForm()
subunit = bcforms.core.Subunit('subunit_a', 2,
   bpforms.ProteinForm().from_str('CAAAAAAAA'))
form_2_b.subunits.append(subunit)
form_2_b.crosslinks.append(bcforms.core.OntologyCrosslink(
   'disulfide', 'subunit_a', 1, 'subunit_a', 1, 1, 2))

# Get the subunits and crosslinks
form_1.subunits[0].id »subunit_a
form_2.crosslinks[0] »<bcforms.core.OntologyCrosslink at 0x7f89c56949e8>

# Get the string representation of a complex
str(form_1_b) »2 * subunit_a + 3 * subunit_b

# Check equality of complexes
form_1_b.is_equal(form_1) »True

# Calculate properties of a complex
form_1.get_structure()[0] »<openbabel.OBMol>
form_1.export('smiles') »C(=O)([C@@H]([NH3+])CS)N...
str(form_1.get_formula()) »C135H240N45O50S5
form_1.get_charge() »5
form_1.get_mol_wt() »3453.9699
```

Box S9. Tutorial for the *BcForms* Python library. This tutorial and a more detailed tutorial are also available at https://sandbox.karrlab.org.

built the alphabets using BeautifulSoup [28], ChemAxon Marvin [11], Open Babel [12], Requests [29], and SQLAlchemy [30]. We described the alphabets in YAML Ain't Markup Language [31], and used ruamel.yaml [32] to parse the alphabets. We used Open Babel and Marvin to implement calculations of properties of macromolecules. We used Marvin to implement the molecular visualizations, and implemented the genomic visualizations using Scalable Vector Graphics (SVG) [33]. We used BioPython [14] to implement methods for importing and exporting *BpForms*-encoded polymers to and from FASTA documents. We implemented the command-line interfaces with Cement [34] and

implemented the REST APIs with Flask-RESTPlus [35]. We implemented the web applications using Zurb Foundation [36] and FancyBox [37].

We deployed the web applications on a virtual private server using Passenger [38].

We used the unittest module [39] to develop over 250 tests to verify *BpForms* and *BcForms*. We used Coverage.py [40] to check that our tests cover over 95% of the code.

We used reStructuredText [41] and Sphinx [42] to generate documentation for *BpForms* and *BcForms*. We used Jupyter [43] to develop interactive tutorials for *BpForms* and *BcForms*.

13. Comparisons with other formats and resources

13.1. Comparison of the *BpForms* grammar with other formats

Several formats, such as the Proteomics Standards Initiative Extended FASTA Format (PEFF) [44] and ProForma [45], have been developed to represent the structure of DNA, RNA, and proteins. In addition, several formats for representing molecular networks, such as BioPAX and SBOL, have limited abilities to represent the structure of DNA, RNA, and proteins.

As described below and summarized in Table S1, we believe that *BpForms* has advantages for omics, systems biology, and synthetic biology research because it abstracts the complete molecular structure of DNA, RNA, and proteins as collections of residues, crosslinks, and nicks; *BpForms* can capture several types of missing information about polymers; *BpForms* is both human and machine-readable; *BpForms* is backward compatible with the IUPAC/IUBMB format; and *BpForms* can be integrated into omics, systems biology, and synthetic biology formats such as BioPAX, CellML, SBML, and SBOL.

Although *BpForms* has several advantages for network research, *BpForms* has two principal limitations. First, unlike the PDB format, *BpForms* cannot represent three-dimensional information. Second, because *BpForms* has a closed schema, *BpForms* is less extensible than formats that have open schemas such as the Chemical Markup Language (CML) [46] and BioPAX.

Consistency in representing DNA, RNA, and proteins. Like the IUPAC/IUBMB format, *BpForms* can represent DNA, RNA, and proteins. In contrast, the MODOMICS nomenclature only represents RNA and the Biological Expression Language (BEL) [54], PEFF, PRO, and ProForma only represent proteins.

We anticipate that *BpForms*' consistent representation of DNA, RNA, and proteins will facilitate the adoption of *BpForms*, as well as facilitate the integration of information about epigenetic, post-transcriptional, and post-translational modification into comprehensive maps, models, and genetic designs.

Concrete representation of the chemical structure of polymers. Like molecular formats such as the International Chemical Identifier (InChI) [49], the Protein Data Bank (PDB) format [53], the Simplified Molecular-Input Line-Entry System (SMILES) [1], and BigSMILES [47], *BpForms* can represent the molecular structure of polymers including NC residues, crosslinks, and nicks. In contrast, BEL, BioPAX, the MODOMICS nomenclature, PEFF, the PRO format, ProForma, and SBOL do not represent the bonding of NC residues or nicks, and only BioPAX has limited abilities to represent crosslinks. Although HELM [48], can represent crosslinks, it does not support high-level semantics as *BpForms* and *BcForms* do via the ontology of crosslinks.

	Format	DNA	RNA	Proteins	Alphabet-defined residues	User-defined residues	Crosslinks	Nicks	3D structure	Concrete semantics	Capture knowledge gaps	Abstract	User-defined alphabets	Open schema	Human-readable	Machine-readable	Software tools	Backward compatible	Composable
Molecules	*BpForms*	✓	✓	✓	✓	✓	✓	✓		✓	✓	✓	✓		✓	✓	✓	✓	✓
	BigSMILES [47]	✓	✓	✓		×	✓	✓		✓		×			×	✓			✓
	HELM [48]	✓	✓	✓	✓	✓	✓	✓		✓		✓	✓		✓	✓	✓		✓
	InChI [49]	✓	✓	✓		×	✓	✓		✓						✓	✓		✓
	IUPAC/IUBMB [50]	✓	✓	✓						✓		✓	✓			✓	✓	✓	✓
	MODOMICS nomenclature [51]		✓		✓					×		✓	✓					✓	✓
	PEFF [44]			✓	✓		×					✓			✓	✓	✓	✓	✓
	PRO proteoform format [52]			✓	✓		×	×			×	✓			✓	×			✓
	ProForma [45]			✓	✓	×					×	✓	✓		×			×	✓
	Protein Data Bank (PDB) format [53]	✓	✓	✓	✓	×	✓	✓	✓	✓		✓				✓	✓		
	SMILES [1]	✓	✓	✓		×	✓	✓		✓						✓	✓		✓
Networks	BEL [54]			✓	✓							✓			✓	✓	✓		✓
	BioPAX [15]	✓	✓	✓	×		✓					✓	✓	✓		✓	✓		
	SBOL [24]	✓	✓	✓								✓	✓	✓		✓	✓		

Table S1. Comparison between *BpForms* and other formats for describing polymers. The 'DNA', 'RNA', and 'Proteins' columns indicate which formats can describe DNA, RNA, and proteins. The 'Alphabet-defined residues' and 'User-defined residues' columns indicate which formats are linked to alphabets and which formats enable users to define residues inline. The 'Crosslinks' and 'Nicks' columns indicate which formats capture crosslinks and nicks. The '3D structure' column indicates which formats describe the three-dimensional structures of polymers. The 'Concrete semantics' column indicates which formats capture the molecular structure of polymers. The 'Capture knowledge gaps' column indicates which formats capture missing information such as the location of a non-canonical residue. The 'Abstract' column indicates which formats represent polymers as sequences of residues and sets of crosslinks. The 'User-defined alphabets' column indicates which formats enable users to define their own alphabets. The 'Open schema' column indicates which formats have open schemas similar to the Resource Description Framework (RDF). The 'Backward compatible' column indicates which formats are compatible with the IUPAC/IUBMB format. The 'Composable' column indicates which formats can be integrated into other formats such as CellML, SBML, and SBOL. Each ✓ indicates a feature of a format; each × indicates a partially-supported feature of a format.

Representation of missing information. Similar to the PRO format, *BpForms* can capture several types of missing information about polymers such as the locations NC residues; the structures, masses, and charges of NC residues; and the locations of crosslinks. In contrast, ProForma can only represent missing knowledge about the structures and masses of residues. BEL, BigSMILES, BioPAX, HELM, InChI, the MODOMICS nomenclature, the PDB format, PEFF, SBOL, and SMILES cannot represent missing knowledge.

We believe that the ability to represent missing knowledge makes *BpForms* well-suited for omics, WC modeling, and whole-genome engineering which need to represent both knowledge and gaps in knowledge.

Human readability: abstraction of chemistry. *BpForms* uses alphabets of residues and an ontology of crosslinks to abstract the structures of polymers. These abstractions make *BpForms*-encoded descriptions of polymers easy to read and write. Furthermore, users can define their own abstractions within descriptions of polymers or define their own alphabet of residues or ontology of crosslinks. This enables *BpForms* to represent newly discovered and synthetic residues. BEL, HELM, the IUPAC/IUBMB format, the MODOMICS nomenclature, PEFF, the PRO format, and ProForma are similarly human-readable.

Although the PDB format uses an alphabet, PDB documents are hard to read and write because the format has limited abilities to abstract residues which do not belong to the alphabet, the format has limited abilities to abstract crosslinks, the format does not abstract nicks, and the format is verbose. Molecular formats such as SMILES are not readable for large molecules such as proteins. BioPAX and SBOL are also difficult to read and write because they are verbose.

Machine-readability: formal grammar. Like BEL, BigSMILES, BioPAX, HELM, InChI, IUPAC/IUBMB, the PDB format, PEFF, SMILES, and SBOL, *BpForms* is machine-readable because it has a formal grammar. We have used this grammar to build software tools for parsing, validating, calculating properties, exporting, and composing *BpForms*-encoded descriptions of polymers into computational workflows. In contrast, the MODOMICS nomenclature, the PRO format, and ProForma are not machine-readable because we are not aware of formal grammars or software tools for these formats.

Backward compatibility with IUPAC/IUBMB and sequence informatics tools. Like the MODOMICS nomenclature, PEFF, and ProForma, *BpForms* maximizes compatibility with sequence informatics tools by generalizing the IUPAC/IUBMB format. As a result, *BpForms* can be integrated into FASTA documents. In contrast, BEL, BigSMILES, BioPAX, HELM, InChI, the PDB format, the PRO format, SBOL, and SMILES are less compatible with sequence informatics tools because they are not backward compatible with the IUPAC/IUBMB format.

Composability with other formats. *BpForms* is compact like other text formats such as BEL, BigSMILES, HELM, the IUPAC/IUBMB format, the MODOMICS nomenclature, PEFF, the PRO format, and ProForma. This makes *BpForms* composable with formats for describing entire pathways, models, and genetic design such as BioPAX, CellML, SBML, and SBOL. In contrast, BioPAX, the PDB format, and SBOL are less suited to integration into other formats because they are verbose.

13.2. Comparison of the *BpForms* alphabets with other resources

Several databases have been developed to help exchange information about NC DNA, RNA, and protein residues. As described below and summarized in Table S2, we believe that the *BpForms* alphabets have advantages for omics, systems biology, and synthetic biology research because they represent DNA, RNA, and proteins; they represent concrete chemical structures and bonding sites; and they are the most comprehensive collections of residues.

Consistency in representing DNA, RNA, and proteins. Like the Protein Data Bank (PDB) Chemical Component Dictionary (CCD) [5], the *BpForms* alphabets represent DNA, RNA, and protein residues. This consistency makes *BpForms* easy to use and facilitates the integration of information, models, and genetic designs that involve DNA, RNA, and proteins. In contrast, DNAmod and REPAIRtoire only represent DNA residues, MODOMICS, and the RNA Modification Database only represent RNA residues, and the Protein Modification Ontology (MOD) and RESID only represent protein residues.

Alphabet	DNA	RNA	Protein	Entire residue	Structure	Bonding	Structural evidence	Biochemical evidence
BpForms	422	378	1,435	✓	✓	✓	✓	✓
DNAmod [6] (verified nucleobases)	58				✓			✓
REPAIRtoire [7] (monophosphates)	34			✓	✓			✓
MODOMICS [51]		172			✓			✓
RNA Modification Database [9]		112			✓			✓
PDB CCD [5] (unambiguous released residues)	373	271	1,095	✓	✓	✓	✓	
Protein Modification Ontology (MOD) [55] (leaves)			1,445	×	×		×	✓
RESID [10]			621		✓			✓
World-wide Monomer Reference Database [56]		348	121		✓	✓		

Table S2. Comparison between *BpForms* and other collections of DNA, RNA, and protein residues. The 'DNA', 'RNA', and 'Protein' columns indicate the numbers of DNA, RNA, and protein residues represented by each resource. The 'Entire residue' column indicates which resources represent entire residues, such as nucleotide monophosphates, rather than parts of residues, such as nucleobases. The 'Structure' column indicates which resources describe the molecular structure of each residue. The 'Bonding' column indicates which resources capture the sites within each residue which can bond with preceding and following residues. The 'Structural evidence' column indicates which resources include residues that have been discovered via structural methods such as x-ray crystallography. The 'Biochemical evidence' column indicates which resources include residues that have been discovered via biochemical methods such as antibodies. Each ✓ indicates a feature of a collection; each × indicates a partially-supported feature of a collection.

Concreteness of chemical semantics. Like the PDB CCD, the *BpForms* alphabets define complete residues and each residue defines a concrete chemical structure and concrete bonding sites with the preceding and following residues. This enables *BpForms* to represent the primary structures of NC polymers. This also enables *BpForms* to capture modifications to the sugar-phosphate backbone of DNA and RNA. In contrast, DNAmod, MODOMICS, and RNA Modification Database have limited abilities to represent NC DNA and RNA because these formats do not represent the sugar-phosphate backbone, and the formats have ambiguous chemical semantics because they do not capture bonding sites; REPAIRtoire and RESID have ambiguous chemical semantics because they do not represent bonding sites; and most MOD entries have ambiguous chemical semantics because they do not define concrete structures or bonding sites.

Breadth of residues from structural and biochemical studies. To make *BpForms* useful for structural biology, omics, systems biology, and synthetic biology, we populated the *BpForms* alphabets with residues that are important for a wide range of research. As a result, the *BpForms* alphabets are the most comprehensive collections of residues. In contrast, the PDB CCD represents fewer residues because it is primarily based on structural biology data and DNAmod, MOD, MODOMICS, REPAIRtoire, the RNA Modification Database, and RESID represent fewer residues because they are mainly based on biochemical data.

Resource	Represents crosslinks	Represents dimers	Concrete semantics	Composable
BpForms	✓		✓	✓
Protein Data Bank (PDB) [53]	×		✓	×
Protein Modification Ontology (MOD) [55]		✓	×	
REPAIRtoire [7]		✓	×	
RESID [10]		✓	✓	
UniProt [19]	✓			

Table S3. Comparison between the *BpForms* crosslinks ontology and other resources that describe crosslinks. The 'Represents crosslinks' and 'Represents dimers' columns indicate which resources describe crosslinks or dimers than contain crosslinks. The 'Concrete semantics' column indicates which resources describe the atoms involved in each crosslink. The 'Composable' column indicates which resources can be composed into chemically-concrete descriptions of macromolecules. Each ✓ indicates a feature of a resource; each × indicates a partially-supported feature of a resource.

13.3. Comparison of the *BpForms* crosslink ontology with other resources

Several resources include information about crosslinks. As illustrated in Table S3, we believe that the *BpForms* crosslink ontology has advantages for omics and systems and synthetic biology research because it concretely represents crosslinks and it is composable with the residues in the *BpForms* alphabets into descriptions of macromolecules. In contrast, REPAIRtoire [7], the Protein Modification Ontology (MOD) [55], and RESID [10] use residues to indirectly represent crosslinks, and the crosslinks in the UniProt controlled vocabulary of posttranslational modifications [19] do not have concrete chemical semantics. As a result, the crosslinks represented by these resources are difficult to compose into macromolecules.

13.4. Comparison of the *BcForms* grammar with other formats

Despite the importance of complexes, only a few formats have been developed to represent complexes. As described below and summarized in Table S4, we believe that *BcForms* has advantages for omics, systems biology, and synthetic biology research because it abstractly represents the primary structure of complexes and it is human-readable, machine-readable, and composable with formats for network research such as CellML and SBML.

Although *BcForms* has several advantages for network research, the *BcForms* grammar has the same disadvantages as the *BpForms* grammar: the grammar cannot represent three-dimensional information and the grammar is less flexible than grammars that have open schemas such as CML.

Representation of the chemical structure of complexes. Like BigSMILES, HELM, InChI, the PDB format, and SMILES, *BcForms* can represent the primary structure of complexes, including NC subunits and interchain crosslinks. In contrast, BioPAX and SBOL have limited abilities to represent crosslinks. We anticipate that the concrete semantics of *BcForms* will facilitate the integration of data, models, and genetic designs that involve complexes.

Format	Concrete semantics	3D structure	Abstract	Open schema	Human-readable	Machine-readable	Software tools	Composable
BcForms	✓		✓		✓	✓	✓	✓
BioPAX [15]			✓	✓		✓	✓	
International Chemical Identifier (InChI) [49]	✓					✓	✓	✓
Protein Data Bank (PDB) format [53]	✓	✓	×			✓	✓	
Synthetic Biology Open Language (SBOL) [24]			✓	✓		✓	✓	
Simplified Molecular-Input Line-Entry System (SMILES) [1]	✓					✓	✓	✓

Table S4. Comparison between *BcForms* and other formats for describing complexes. The 'Concrete semantics' column indicates which formats capture the molecular structures of complexes. The 'Abstract' column indicates which formats abstract the structures of complexes as combinations of subunits and crosslinks. The '3D structure' column indicates which formats represent the three-dimensional structures of complexes. The 'Open schema' indicates which formats have open schemas similar to RDF. The 'Composable' column indicates which formats can be integrated into other formats such as CellML, SBML, and SBOL. Each ✓ indicates a feature of a format; each × indicates a partially-supported feature of a format.

BpForms id	PDB CCD id	Name	PDB entries	Proteins	Absence from *E. coli*
AA0030	HYP	4-hydroxyproline	239	Collagen and plant walls	[57–59]
AA0317	HIC	4-methyl-histidine	121	Actin and myosin	[60, 61]
AA0070	MEN	N-methyl asparagine	57	Antennae of photosystem II	[62–64]
FVA	FVA	N-formyl-L-valine	23	Gramicidin	
6V1	6V1		10		
TQQ	TQQ		8	Aromatic amine dehydrogenase	
TRX	TRX	6-hydroxytryptophan	6	RNA polymerase II	
PSW	PSW	3-(sulfanylselanyl)-L-alanine	5		

Table S5. The most common constraints on transforming proteins into *E. coli* due to missing post-translational machinery learned from the PDB with *BpForms*. We identified residues which cannot be synthesized by *E. coli* by using *BpForms* to analyze the PDB, and we confirmed the absence of the most frequent residues from *E. coli* via the literature. This information could be used to constrain the design of novel strains of *E. coli*. For example, genetic designs based on *E. coli* should not include photosystem II, or such designs should also include phycobiliprotein asparagine methyltransferase CpcM [63].

Abstraction, human readability, and composability. Similar to BioPAX, and SBOL, *BcForms* abstracts complexes as sets of subunits and crosslinks. This makes *BcForms* human-readable and composable with formats for systems biology and synthetic biology research such as CellML, SBML, and SBOL. By comparison, BigSMILES and HELM are less human-readable and not designed to capture subunits that are not connected by crosslinks, InChI and SMILES are less human-readable, and the PDB format is both less human-readable and less composable.

14. Additional information for case studies in the main text

Table S5 provides additional information for the synthetic biology case study discussed in the main text.

15. Glossary

Alphabet of residues A collection that maps the code of each residue to its molecular structure and a list of its atoms that can bond with preceding and following residues.

Cap A residue which can only be located at the right end of a polymer because it can only bond with preceding residues, or a residue which can only be located at the left end of a polymer because it can only bond with following residues. Examples of caps include the 5' RNA caps 7-methylguanylate, 7-dimethylguanylate, and 7-trimethylguanylate, which are vital for the stability of eukaryotic mRNA. Because caps are similar to residues, *BpForms* represents caps as residues (which can only bond with preceding or following residues).

Crosslink A covalent bond between non-adjacent residues, such as a disulfide bond between the sulfur atoms of two cysteine amino acids.

Grammar A structured text format for describing information such as the molecular structure of a polymer or complex. Additional examples include the InChI and SMILES formats.

Intra-chain crosslink A crosslink between two residues in the same polymer.

Inter-chain crosslink A crosslink between two residues in different polymers.

Nick Absence of an inter-residue bond between successive residues.

Non-canonical residue A residue that is not one of the four standard DNA nucleic acids (A, C, G, T), four standard RNA nucleic acids (A, C, G, U), or twenty standard protein amino acids.

Ontology of crosslinks A collection that maps the id of each crosslink to a list of the atoms which participate in the crosslink.

Residue A component, such as a nucleotide monophosphate or amino acid, of the sequence of a polymer.

16. Acronyms

APT Advanced Package Tool [65]

BEL Biological Expression Language [54]

BNF Backus-Naur Form

CML Chemical Markup Language [46]

COMBINE Computational Modeling in Biology Network [66]

EBNF Extended Backus-Naur Form [3]

InChI International Chemical Identifier [49]

MOD Protein Modification Ontology [55]

NC Non-canonical

PDB Protein Data Bank [53]

PDB CCD PDB Chemical Component Dictionary [5]

PRO Protein Ontology [52]

RDF Resource Description Framework

REST Representational State Transfer

SBML Systems Biology Markup Language [18]

SBOL Synthetic Biology Open Language [24]

SMILES Simplified Molecular-Input Line-Entry System [1]

SVG Scalable Vector Graphics [33]

WC Whole-cell [21, 22]

References

[1] Weininger, D.: SMILES, a chemical language and information system. 1. introduction to methodology and encoding rules. J. Chem. Inform. Comp. Sci. **28**(1), 31–36 (1988)

[2] Hastings, J., Owen, G., Dekker, A., Ennis, M., Kale, N., Muthukrishnan, V., Turner, S., Swainston, N., Mendes, P., Steinbeck, C.: ChEBI in 2016: Improved services and an expanding collection of metabolites. Nucleic Acids Res. **44**(D1), 1214–1219 (2015)

[3] Extended Backus-Naur Form. https://en.wikipedia.org/wiki/Extended_Backus-Naur_form. Accessed 02 August 2019.

[4] Lark – a modern parsing library for Python. https://lark-parser.readthedocs.io. Accessed 20 June 2019.

[5] Westbrook, J.D., Shao, C., Feng, Z., Zhuravleva, M., Velankar, S., Young, J.: The Chemical Component Dictionary: complete descriptions of constituent molecules in experimentally determined 3d macromolecules in the Protein Data Bank. Bioinformatics **31**(8), 1274–1278 (2014)

[6] Sood, A.J., Viner, C., Hoffman, M.M.: DNAmod: the DNA modification database. J. Cheminform. **11**(1), 30 (2019)

[7] Milanowska, K., Krwawicz, J., Papaj, G., Kosiński, J., Poleszak, K., Lesiak, J., Osińska, E., Rother, K., Bujnicki, J.M.: REPAIRtoire–a database of DNA repair pathways. Nucleic Acids Res. **39**(suppl_1), 788–792 (2010)

[8] Machnicka, M.A., Milanowska, K., Osman Oglou, O., Purta, E., Kurkowska, M., Olchowik, A., Januszewski, W., Kalinowski, S., Dunin-Horkawicz, S., Rother, K.M., *et al.*: MODOMICS: a database of RNA modification pathways–2013 update. Nucleic Acids Res. **41**(D1), 262–267 (2012)

[9] Cantara, W.A., Crain, P.F., Rozenski, J., McCloskey, J.A., Harris, K.A., Zhang, X., Vendeix, F.A., Fabris, D., Agris, P.F.: The RNA Modification Database, RNAMDB: 2011 update. Nucleic Acids Res. **39**(suppl_1), 195–201 (2010)

[10] Garavelli, J.S.: The RESID Database of Protein Modifications as a resource and annotation tool. Proteomics **4**(6), 1527–1533 (2004)

[11] Marvin. https://chemaxon.com/products/marvin. Accessed 02 August 2019.

[12] O'Boyle, N.M., Guha, R., Willighagen, E.L., Adams, S.E., Alvarsson, J., Bradley, J.-C., Filippov, I.V., Hanson, R.M., Hanwell, M.D., Hutchison, G.R., *et al.*: Open data, open source and open standards in chemistry: the Blue Obelisk five years on. J. Cheminform. **3**(1), 37 (2011)

[13] Pearson, W.R.: Rapid and sensitive sequence comparison with FASTP and FASTA. Methods Enzymol. **183**, 63–98 (1990)

[14] Cock, P.J., Antao, T., Chang, J.T., Chapman, B.A., Cox, C.J., Dalke, A., Friedberg, I., Hamelryck, T., Kauff, F., Wilczynski, B., *et al.*: Biopython: freely available Python tools for computational molecular biology and bioinformatics. Bioinformatics **25**(11), 1422–1423 (2009)

[15] Demir, E., Cary, M.P., Paley, S., Fukuda, K., Lemer, C., Vastrik, I., Wu, G., D'eustachio, P., Schaefer, C., Luciano, J., *et al.*: The BioPAX community standard for pathway data sharing. Nat. Biotechnol. **28**(9), 935–942 (2010)

[16] Bergmann, F.T., Rodriguez, N., Le Novère, N.: COMBINE archive specification version 1. J. Integr. Bioinform. **12**(2), 104–118 (2015)

[17] Cuellar, A., Hedley, W., Nelson, M., Lloyd, C., Halstead, M., Bullivant, D., Nickerson, D., Hunter, P., Nielsen, P.: The CellML 1.1 specification. J. Integr. Bioinform. **12**(2), 4–85 (2015)

[18] Hucka, M., Bergmann, F.T., Dräger, A., Hoops, S., Keating, S.M., Le Novère, N., Myers, C.J., Olivier, B.G., Sahle, S., Schaff, J.C., et al.: The Systems Biology Markup Language (SBML): language specification for level 3 version 2 core. J. Integr. Bioinform. **15**(1) (2018)

[19] UniProt Consortium, *et al.*: UniProt: the universal protein knowledgebase. Nucleic Acids Res. **45**(D1), 158–169 (2017)

[20] Tyson, J.J.: Modeling the cell division cycle: cdc2 and cyclin interactions. Proc. Natl. Acad. Sci. U. S. A. **88**(16), 7328–7332 (1991)

[21] Karr, J.R., Sanghvi, J.C., Macklin, D.N., Gutschow, M.V., Jacobs, J.M., Bolival Jr, B., Assad-Garcia, N., Glass, J.I., Covert, M.W.: A whole-cell computational model predicts phenotype from genotype. Cell **150**(2), 389–401 (2012)

[22] Goldberg, A.P., Szigeti, B., Chew, Y.H., Sekar, J.A., Roth, Y.D., Karr, J.R.: Emerging whole-cell modeling principles and methods. Curr. Opin. Biotechnol. **51**, 97–102 (2018)

[23] Wang, C.-C., Cirit, M., Haugh, J.M.: PI3K-dependent cross-talk interactions converge with Ras as quantifiable inputs integrated by erk. Mol. Syst. Biol. **5**(1) (2009)

[24] Cox, R.S., Madsen, C., McLaughlin, J.A., Nguyen, T., Roehner, N., Bartley, B., Beal, J., Bissell, M., Choi, K., Clancy, K., et al.: Synthetic Biology Open Language (SBOL) version 2.2.0. J. Integr. Bioinform. **15**(1) (2018)

[25] SBOL SEP 033 – Concrete descriptions of non-canonical DNA, RNA, and proteins. https://github.com/SynBioDex/SEPs/blob/master/sep_033.md. Accessed 20 June 2019.

[26] PyPI: The Python Package Index. https://pypi.org. Accessed 20 June 2019.

[27] Python. https://python.org/. Accessed 02 August 2019.

[28] Beautiful Soup. https://www.crummy.com/software/BeautifulSoup/. Accessed 02 August 2019.

[29] Requests: HTTP for humans. https://2.python-requests.org. Accessed 02 August 2019.

[30] SQLAlchemy – The database toolkit for Python. https://www.sqlalchemy.org/. Accessed 02 August 2019.

[31] YAML: YAML Ain't Markup Language. https://yaml.org. Accessed 02 August 2019.

[32] ruamel.yaml. https://yaml.readthedocs.io. Accessed 02 August 2019.

[33] Scalable Vector Graphics (SVG). https://www.w3.org/Graphics/SVG/. Accessed 02 August 2019.

[34] Cement framework. https://builtoncement.com/. Accessed 02 August 2019.

[35] Flask-RESTPlus. https://flask-restplus.readthedocs.io. Accessed 02 August 2019.

[36] Foundation – The most advanced responsive front-end framework in the world. https://foundation.zurb.com. Accessed 02 August 2019.

[37] FancyBox – Fancy jQuery lightbox alternative. http://fancybox.net. Accessed 02 August 2019.

[38] Passenger. https://www.phusionpassenger.com. Accessed 02 August 2019.

[39] unittest unit testing framework. https://docs.python.org/3/library/unittest.html. Accessed 02 August 2019.

[40] Coverage.py. https://coverage.readthedocs.io. Accessed 02 August 2019.

[41] reStructuredText. http://docutils.sourceforge.net/rst.html. Accessed 02 August 2019.

[42] Sphinx – Python documentation generator. http://www.sphinx-doc.org. Accessed 02 August 2019.

[43] Jupyter. https://jupyter.org. Accessed 02 August 2019.

[44] Binz, P.-A., Shofstahl, J., Vizcaíno, J.A., Barsnes, H., Chalkley, R.J., Menschaert, G., Alpi, E., Clauser, K., Eng, J.K., Lane, L., et al.: Proteomics Standards Initiative Extended FASTA Format (PEFF). J. Proteome Res. (2019)

[45] LeDuc, R.D., Schwämmle, V., Shortreed, M.R., Cesnik, A.J., Solntsev, S.K., Shaw, J.B., Martin, M.J., Vizcaino, J.A., Alpi, E., Danis, P., *et al.*: ProForma: a standard proteoform notation. J. Proteome Res. **17**(3), 1321–1325 (2018)

[46] Murray-Rust, P., Townsend, J.A., Adams, S.E., Phadungsukanan, W., Thomas, J.: The semantics of Chemical Markup Language (CML): dictionaries and conventions. J. Cheminform. **3**(1), 43 (2011)

[47] Lin, T.-S., Coley, C.W., Mochigase, H., Beech, H.K., Wang, W., Wang, Z., Woods, E., Craig, S.L., Johnson, J.A., Kalow, J.A., *et al.*: BigSMILES: a structurally-based line notation for describing macromolecules. ACS Cent Sci **5**(9), 1523–1531 (2019)

[48] Zhang, T., Li, H., Xi, H., Stanton, R.V., Rotstein, S.H.: HELM: a hierarchical notation language for complex biomolecule structure representation. ACS Publications (2012)

[49] Heller, S.R., McNaught, A., Pletnev, I., Stein, S., Tchekhovskoi, D.: InChI, the IUPAC international chemical identifier. J. Cheminform. **7**(1), 23 (2015)

[50] Leonard, S.A.: IUPAC/IUB single-letter codes within nucleic acid and amino acid sequences. Curr. Protoc. Bioinformatics (1), 1 (2003)

[51] Boccaletto, P., Machnicka, M.A., Purta, E., Piątkowski, P., Bagiński, B., Wirecki, T.K., de Crécy-Lagard, V., Ross, R., Limbach, P.A., Kotter, A., *et al.*: MODOMICS: a database of rna modification pathways. 2017 update. Nucleic Acids Res. **46**(D1), 303–307 (2017)

[52] Natale, D.A., Arighi, C.N., Blake, J.A., Bona, J., Chen, C., Chen, S.-C., Christie, K.R., Cowart, J., D'Eustachio, P., Diehl, A.D., *et al.*: Protein Ontology (PRO): enhancing and scaling up the representation of protein entities. Nucleic Acids Res. **45**(D1), 339–346 (2017)

[53] Westbrook, J.D., Fitzgerald, P.: The PDB format, mmCIF, and other data formats. In: Bourne, P.E., Weissig, H. (eds.) Structural Bioinformatics vol. 44, pp. 161–179. Wiley Online Library, ??? (2003). Chap. 8

[54] Fluck, J., Madan, S., Ansari, S., Karki, R., Rastegar-Mojarad, M., Catlett, N.L., Hayes, W., Szostak, J., Hoeng, J., Peitsch, M., *et al.*: Training and evaluation corpora for the extraction of causal relationships encoded in biological expression language (BEL). Database **2016**(pii), 113 (2016)

[55] Montecchi-Palazzi, L., Beavis, R., Binz, P.-A., Chalkley, R.J., Cottrell, J., Creasy, D., Shofstahl, J., Seymour, S.L., Garavelli, J.S.: The PSI-MOD community standard for representation of protein modification data. Nat. Biotechnol. **26**(8), 864–866 (2008)

[56] Milton, J., Zhang, T., Bellamy, C., Swayze, E., Hart, C., Weisser, M., Hecht, S., Rotstein, S.: HELM software for biopolymers. J Chem Inf Model **57**(6), 1233–1239 (2017)

[57] Pinkas, D.M., Ding, S., Raines, R.T., Barron, A.E.: Tunable, post-translational hydroxylation of collagen domains in Escherichia coli. ACS Chem. Biol. **6**(4), 320–324 (2011)

[58] An, B., Kaplan, D.L., Brodsky, B.: Engineered recombinant bacterial collagen as an alternative collagen-based biomaterial for tissue engineering. Front. Chem. **2**, 40 (2014)

[59] Yi, Y., Sheng, H., Li, Z., Ye, Q.: Biosynthesis of trans-4-hydroxyproline by recombinant strains of Corynebacterium glutamicum and Escherichia coli. BMC Biotechnol. **14**(1), 44 (2014)

[60] Kwiatkowski, S., Seliga, A.K., Vertommen, D., Terreri, M., Ishikawa, T., Grabowska, I., Tiebe, M., Teleman, A.A., Jagielski, A.K., Veiga-da-Cunha, M., *et al.*: SETD3 protein is the actin-specific histidine N-methyltransferase. Elife **7**, 37921 (2018)

[61] Xiao, H., Peters, F.B., Yang, P.-Y., Reed, S., Chittuluru, J.R., Schultz, P.G.: Genetic incorporation of histidine derivatives using an engineered pyrrolysyl-tRNA synthetase. ACS Chem. Biol. **9**(5), 1092–1096 (2014)

[62] Klotz, A., Glazer, A.N.: gamma-n-methylasparagine in phycobiliproteins. occurrence, location, and biosynthesis. J. Biol. Chem. **262**(36), 17350–17355 (1987)

[63] Shen, G., Leonard, H.S., Schluchter, W.M., Bryant, D.A.: CpcM posttranslationally methylates asparagine-71/72 of phycobiliprotein beta subunits in Synechococcus sp. strain PCC 7002 and Synechocystis sp. strain PCC 6803. J. Bacteriol. **190**(14), 4808–4817 (2008)

[64] Scheer, H., Zhao, K.-H.: Biliprotein maturation: the chromophore attachment. Mol. Microbiol. **68**(2), 263–276 (2008)

[65] Apt. https://help.ubuntu.com/lts/serverguide/apt.html. Accessed 20 June 2019.

[66] Hucka, M., Nickerson, D.P., Bader, G.D., Bergmann, F.T., Cooper, J., Demir, E., Garny, A., Golebiewski, M., Myers, C.J., Schreiber, F., *et al.*: Promoting coordinated development of community-based information standards for modeling in biology: the COMBINE initiative. Front. Bioeng. Biotechnol. **3**, 19 (2015)
