# Supplementary material for: BpForms and BcForms: a toolkit for concretely describing non-canonical polymers and complexes to facilitate global biochemical networks
Source: Genome Biol. 2020 May 18;21:117. doi: 10.1186/s13059-020-02025-z (PMC7236495; doi:10.1186/s13059-020-02025-z)
Supplement: Supplementary file 2 — Additional file 2 Review history. [file 13059_2020_2025_MOESM2_ESM.docx]

Review History

**First round of review**

**Reviewer 1**

**Were you able to assess all statistics in the manuscript, including the appropriateness of statistical tests used?**

Yes: There is no statistics in the manuscript.

**Were you able to directly test the methods?**

Yes

**Comments to author:**

The authors present a toolkit called "bpForms and bcForms" that offers human-readable textual descriptions as well as molecular visualizations of non-canonical polymers and complexes, including RNA, DNA, proteins and complexes. The descriptions provided by the toolkit cover residues, caps, crosslinks and nicks within polymers and complexes. The textual representations are based on a compiled alphabet of residues and atoms, and a grammar for describing molecular bonds between these atoms within and across residues. The toolkit also provides calculations of several polymer physiochemical properties. The toolkit is very well described in the paper with several examples illustrating its features, and is also very well documented online. The authors offer the toolkit as a web application, REST APIs, command-line programs and python libraries.

The authors have put much effort into providing their toolkit in a user-friendly way. Although most of the features provided by this toolkit are also offered either fully or partially by other existing toolkits and databases, its main advantage is that it integrates multiple features that are not offered all together simultaneously by another existing toolkit alone. One main feature offered by bpForms and bcForms is the ability for users to define new residues and alphabets, the textual representation of polymers and complexes in a human readable form, and open schema.

I can see how this toolkit can be very helpful for researchers in different areas of molecular biology and medicine. However, I have one major comment. The authors say that the toolkit offers 3D structures of complexes. They do provide somewhat 3D visualizations of polymers as well as human-readable descriptions of molecular bonds, a feature that is also found in other existing tools. However, they do not provide the 3D coordinates of the atoms in the structure, which I think reduces its power as a structural biology tool. I think the toolkit would be more powerful if 3D coordinates are included in the structural attributes of each atom, when possible. Alternatively, the authors may add an option to output the structure in a "PDB-friendly" format allowing the user to retrieve 3D coordinates from PDB for their polymer with minimum effort when such coordinates are available.

**Reviewer 2**

**Were you able to assess all statistics in the manuscript, including the appropriateness of statistical tests used?**

Yes: There is no statistical analysis to assess.

**Were you able to directly test the methods?**

Yes

**Comments to author:**

This is a very well written manuscript describing novel methods for unambiguously defining the structure of macromolecules in a way that is suitable for many domains of application. This is an especially important issue to address as detailed descriptions of these macromolecules are not well defined in existing tools, standards, and ontologies - making it difficult to reproduce or reuse any particular study with confidence.

While the authors are particularly interested in the application of these methods in the holistic understanding of whole cell behaviour, they clearly describe the advantages of their methods across a broad spectrum of application domains and compare to existing alternative solutions. Furthermore they provide software support that enables their approach to interchange with these existing solutions where possible, ensuring that users are able to make use of familiar tools as needed in their existing workflows. The authors are, furthermore, actively reaching out to various domain-specific communities to help adopt their methods and tools to these specific domains. This is great to see.

It is also great to see the extensive set of case studies presented on the manuscript and detailed further in the supplemental material and associated websites. I believe this demonstrates the broad applicability of the BpForms-BcForms toolkit indicating a broad appeal of this manuscript and toolkit. The web applications provided on the associated websites makes it easy for readers to "have a play" to test out the toolkits and explore the capabilities, including the examples covered in the manuscript.

An issue that arose in reviewing this manuscript is that there is often insufficient detail provided in the manuscript with the reader referred to the supplemental material to get the full information required. When dealing with PDFs this is a tedious process, perhaps links direct to the corresponding information on the website would help improve readability? But this would also have to consider the presumably continual development of the toolkits themselves.

The web applications are straightforward to use. I was unable to install the Python packages from PyPI (windows 10, python 3.8.0) - but this seemed to be an issue with the dependencies that could likely be solved by using the docker image (but I did not test this).

Minor suggested corrections:

* pg 7, line 12: I wonder if the authors have considered how to manage user-defined custom ontology terms and the impact that has on interoperabilty and exchange between tools and/or users?

* Similarly, have the authors considered how to handle the extension, updates, corrections, etc., to the defined alphabets over time? Presumably tied to the version of the toolkits and migrating between them?

* page 8, line 13: Would be nice to include (perhaps in the supplemental data?) information on how the authors intend to "...work with the community to curate..."

* Is there a release note or similar that could be linked to in support of the error correction in PRO? This is a very compelling demonstration of the utility of the BpForms-BcForms toolkit and would be great to see the PRO point of view on this.

* page 15 and 16: Git should likely be Github - not aware of Git itself supporting pull requests.

* page 16, line 10: I very much encourage the authors to develop a registry of alphabets!

* page 19, line 4: Given the number of repositories under the Karrlab on Github, it would be useful to have a direct link to the appropriate repositories.

* page 19, line 21: it is not clear which of the available docker images serves this purpose.

* page 19, line 28: it would be useful to tidy up the versions available. The manuscript mentions BpForms 0.0.10 and BcForms 0.0.3, which aligns with the releases on github, but docs.karrlab,org have 0.0.9 and 0.0.2, respectively, as the latest versions available.

**Authors Response**

**Point-by-point responses to the reviewers’ comments:**

Reviewer #1

The authors present a toolkit called "bpForms and bcForms" that offers human-readable textual descriptions as well as molecular visualizations of non-canonical polymers and complexes, including RNA, DNA, proteins and complexes. The descriptions provided by the toolkit cover residues, caps, crosslinks and nicks within polymers and complexes. The textual representations are based on a compiled alphabet of residues and atoms, and a grammar for describing molecular bonds between these atoms within and across residues. The toolkit also provides calculations of several polymer physiochemical properties. The toolkit is very well described in the paper with several examples illustrating its features, and is also very well documented online. The authors offer the toolkit as a web application, REST APIs, command-line programs and python libraries.

The authors have put much effort into providing their toolkit in a user-friendly way. Although most of the features provided by this toolkit are also offered either fully or partially by other existing toolkits and databases, its main advantage is that it integrates multiple features that are not offered all together simultaneously by another existing toolkit alone. One main feature offered by bpForms and bcForms is the ability for users to define new residues and alphabets, the textual representation of polymers and complexes in a human readable form, and open schema.

*Thank you for your encouraging feedback.*

I can see how this toolkit can be very helpful for researchers in different areas of molecular biology and medicine. However, I have one major comment. The authors say that the toolkit offers 3D structures of complexes. They do provide somewhat 3D visualizations of polymers as well as human-readable descriptions of molecular bonds, a feature that is also found in other existing tools. However, they do not provide the 3D coordinates of the atoms in the structure, which I think reduces its power as a structural biology tool. I think the toolkit would be more powerful if 3D coordinates are included in the structural attributes of each atom, when possible. Alternatively, the authors may add an option to output the structure in a "PDB-friendly" format allowing the user to retrieve 3D coordinates from PDB for their polymer with minimum effort when such coordinates are available.

BpForms and BcForms do not capture three-dimensional information. This is stated in the third paragraph of Section 13.1 of Additional file 1 ("... BpForms cannot represent three-dimensional information") and in Table S1. (The previous version of Table S1 mistakenly had a check in the wrong column, which mistakenly suggested that BpForms captures 3-dimensional information; we have corrected this. We apologize for the confusion.)

*We have not designed BpForms and BcForms to capture three-dimensional information for several reasons:*

*●	BpForms and BcForms target biochemical and genomics research where three-dimensional information is often not available.*

*●	In our opinion, three-dimensional information is well-captured by the PDB format.*

*●	The inclusion of three-dimensional information would make the descriptions of macromolecules less concise and human readable, which we believe are essential features of BpForms/BcForms.*

*●	Because the three dimensional coordinate of each atom depends on its context within a whole molecule, three-dimensional information is inconsistent with the abstractions used by BpForms/BcForms. In order to maintain the abstractions used by BpForms/BcForms, structural information would need to be represented in a separate layer, similar to how the PDB format uses SEQRES records for the residue sequence and ATOM records for three-dimensional coordinates. This would lead to a format that resembles the PDB format. In our opinion, this is unnecessary because a suitable format (PDB) already exists.*

*Rather than embedding three-dimensional coordinates into BpForms/BcForms, instead we recommend that structural biology users embed BpForms/BcForms into REMARK records in PDB files. Basically, we suggest using BpForms/BcForms to annotate the semantic meaning of the ATOM and SEQRES records in PDB files. While PDB files already contain SEQRES records, these comments do not completely describe the atoms and bonds of a molecule. This is described on the BpForms website (https://www.bpforms.org/#pdb).*

Reviewer #2

This is a very well written manuscript describing novel methods for unambiguously defining the structure of macromolecules in a way that is suitable for many domains of application. This is an especially important issue to address as detailed descriptions of these macromolecules are not well defined in existing tools, standards, and ontologies - making it difficult to reproduce or reuse any particular study with confidence.

While the authors are particularly interested in the application of these methods in the holistic understanding of whole cell behaviour, they clearly describe the advantages of their methods across a broad spectrum of application domains and compare to existing alternative solutions. Furthermore they provide software support that enables their approach to interchange with these existing solutions where possible, ensuring that users are able to make use of familiar tools as needed in their existing workflows. The authors are, furthermore, actively reaching out to various domain-specific communities to help adopt their methods and tools to these specific domains. This is great to see.

It is also great to see the extensive set of case studies presented on the manuscript and detailed further in the supplemental material and associated websites. I believe this demonstrates the broad applicability of the BpForms-BcForms toolkit indicating a broad appeal of this manuscript and toolkit. The web applications provided on the associated websites makes it easy for readers to "have a play" to test out the toolkits and explore the capabilities, including the examples covered in the manuscript.

*Thank you for your encouraging note. We agree that there is an emerging need for more chemical precision across subsets of molecular biology, genomics, systems biology, and synthetic biology due to advancements such as methylation sequencing and top-down proteomics and increasing desire to merge data. For this reason, and because whole-cell modeling would benefit for more precisely annotated data, we have tried to make BpForms/BcForms useful to a range of research, including adding features that we believe are needed by specific communities.*

An issue that arose in reviewing this manuscript is that there is often insufficient detail provided in the manuscript with the reader referred to the supplemental material to get the full information required. When dealing with PDFs this is a tedious process, perhaps links direct to the corresponding information on the website would help improve readability? But this would also have to consider the presumably continual development of the toolkits themselves.

*Thanks for the feedback. We also prefer online documentation because the format is more conducive to technical details, it supports more interaction, and we can update it as we improve BpForms/BcForms. For this reason, the information in the PDF is also available online at https://bpforms.org, https://bcforms.org, https://sandbox.karrlab.org, and https://docs.karrlab.org. The online version is presented a little differently because it doesn't assume that the reader has read the article. The online version is also largely separated into guides for users and more detailed guides for developers. We included a PDF version of the documentation for readers who prefer PDFs.*

*We have made a few changes to make the two versions of the documentation easier to follow:*

*●	Added links to the online versions of the documentation from the description of Additional file 1 in the main text.*

*●	Added an introduction at the beginning of Additional File 1 that states where the same information is available online.*

*●	Copied the content that was only in the PDF to the online documentation.*

*●	Created Jupyter notebook versions of the boxes with Python code in Additional File 1 (Boxes S8 and S9).*

*●	Added links to the online versions of the content in the boxes in Additional File 1 to their captions.*

The web applications are straightforward to use. I was unable to install the Python packages from PyPI (windows 10, python 3.8.0) - but this seemed to be an issue with the dependencies that could likely be solved by using the docker image (but I did not test this).

*Thank you for the feedback. We have added more detailed installation instructions to the documentation, including a minimal Dockerfile, and additional links to the documentation from https://bpforms.org and https://bcforms.org. We would gladly help others install the tools. We encourage potential users to ask for help via GitHub issues or email.*

*We have also reduced the requirements by formally making the non-essential dependencies optional, and clarifying how to optionally install these dependencies. This includes the only proprietary dependency, ChemAxon Marvin, which is free to academic users and only required to calculate major protonation states and generate molecular diagrams. The core functionality of BpFormsand BcForms doesn't require any proprietary dependencies.*

Minor suggested corrections:

●	pg 7, line 12: I wonder if the authors have considered how to manage user-defined custom ontology terms and the impact that has on interoperabilty and exchange between tools and/or users?

*This is an interesting issue. We discussed this a bit when we were deciding whether or not to support inline residues and custom alphabets. It is possible to interoperate between ontologies and between tools because BpForms and BcForms represent concrete chemical structures (i.e. it is possible to compare molecules represented with different ontologies or formats by converting each description to a common molecular format such as InChI). We have held off implementing a solution for this because we feel that this is an issue for the community, and we would prefer to get input from the community on how the community prefers to address this. We added discussion about this to the online documentation (https://docs.karrlab.org/bpforms/interoperability.html) and we added a little more information to Section 3.1.*

●	Similarly, have the authors considered how to handle the extension, updates, corrections, etc., to the defined alphabets over time? Presumably tied to the version of the toolkits and migrating between them?

*This is also an interesting question for the future. We've held off addressing this because we would like to get input from the community. We added discussion about this to the online documentation (https://docs.karrlab.org/bpforms/version-management.html). We also added a little more information to the end of Section 3.1.*

●	page 8, line 13: Would be nice to include (perhaps in the supplemental data?) information on how the authors intend to "...work with the community to curate..."

*This is another interesting point of discussion for going forward. First of all, we want to make it easy for users to contribute, and we don't want to unilaterally dictate the ontologies. For now, while the user base is small, our strategy is to (a) pull the community by merging all major existing ontologies and (b) encourage the community to push our ontologies by contributing in whatever way they are comfortable (preferably GitHub pull requests or issues). To make this easy, we have created template GitHub issues for modifying residues, creating residues, and adding alphabets. For now, we are hesitant to require more formal proposals for contributions because we don't want to create any unnecessary barriers that might discourage people. As the community grows, it would likely be helpful to formalize the process for contributing to the ontologies. This could include collecting potential contributions via GitHub pull requests and having volunteer editors review the requests and periodically solicit votes on the requests. This is the model that SBOL uses. This is discussed in the online documentation (http://docs.karrlab.org/bpforms/contributing.html). We added information about contributing to the ontologies to Section 8 of Additional File S1.*

●	Is there a release note or similar that could be linked to in support of the error correction in PRO? This is a very compelling demonstration of the utility of the BpForms-BcForms toolkit and would be great to see the PRO point of view on this.

*These corrections will be incorporated into the next release (60.0), likely in the Spring of this year. The corrections will be noted in the release notes, which will be available at https://proconsortium.org/download/ in a file named `pro_release_note.txt`. We added a sentence about this to Section 2.3.*

●	page 15 and 16: Git should likely be Github - not aware of Git itself supporting pull requests.

*We corrected this in the main text and Additional file 1.*

●	page 16, line 10: I very much encourage the authors to develop a registry of alphabets! This would likely be useful because (a) we think different communities may prefer to use different ontologies and (b) it's not necessary to force everyone to use one ontology since descriptions of molecules can be compared across ontologies at the chemical level. This could be implemented as a custom website or as a GitHub repository. Because this is an issue for the community, we would prefer to get input from the community and then implement whatever the community agrees to.

●	page 19, line 4: Given the number of repositories under the Karrlab on Github, it would be useful to have a direct link to the appropriate repositories.

*We replaced the top-level link with two links to the BpForms and BcForms repositories.*

●	page 19, line 21: it is not clear which of the available docker images serves this purpose. We replaced this with a link to a new Dockerfile for building a minimal image with BpForms and BcForms. Users can use this to build their own images using their own licenses for ChemAxon Marvin, which are freely available to academic users.

●	page 19, line 28: it would be useful to tidy up the versions available. The manuscript mentions BpForms 0.0.10 and BcForms 0.0.3, which aligns with the releases on github, but docs.karrlab,org have 0.0.9 and 0.0.2, respectively, as the latest versions available.

*We fixed this bug with how https://docs.karrlab.org forwards to the latest version of the documentation of each package. The latest versions are now 0.0.15 and 0.0.8. These are tagged on GitHub, posted to PyPI, and noted in the manuscript. https://docs.karrlab.org now correctly points to the documentation for these versions. We also updated the websites and https://sandbox.karrlab.orgto the latest versions.*

**Second round of review:**

**Reviewer 2**

I thank the authors for their detailed responses to my review comments and for addressing all points in the revised manuscript (and supplemental material and associated web repositories/sites).
